# Evaluating changes in attractor sets under small network perturbations to infer reliable microbial interaction networks from abundance patterns

**DOI:** 10.1093/bioinformatics/btaf095

**Published:** 2025-03-01

**Authors:** Jyoti Jyoti, Marc-Thorsten Hütt

**Affiliations:** School of Science, Constructor University, Bremen 28759, Germany; School of Science, Constructor University, Bremen 28759, Germany

## Abstract

**Motivation:**

Inferring microbial interaction networks from microbiome data is a core task of computational ecology. An avenue of research to create reliable inference methods is based on a stylized view of microbiome data, starting from the assumption that the presences and absences of microbiomes, rather than the quantitative abundances, are informative about the underlying interaction network. With this starting point, inference algorithms can be based on the notion of attractors (asymptotic states) in Boolean networks. Boolean network framework offers a computationally efficient method to tackle this problem. However, often existing algorithms operating under a Boolean network assumption, fail to provide networks that can reproduce the complete set of initial attractors (abundance patterns). Therefore, there is a need for network inference algorithms capable of reproducing the initial stable states of the system.

**Results:**

We study the change of attractors in Boolean threshold dynamics on signed undirected graphs under small changes in network architecture and show, how to leverage these relationships to enhance network inference algorithms. As an illustration of this algorithmic approach, we analyse microbial abundance patterns from stool samples of humans with inflammatory bowel disease (IBD), with colorectal cancer and from healthy individuals to study differences between the interaction networks of the three conditions. The method reveals strong diversity in IBD interaction networks. The networks are first partially deduced by an earlier inference method called ESABO, then we apply the new algorithm developed here, EDAME, to this result to generate a network that comes nearest to satisfying the original attractors.

**Availability and implementation:**

Implementation code is freely available at https://github.com/Jojo6297/edame.git.

## 1 Introduction

Studying microbial communities via high-throughput techniques has provided deep insight into diverse fields, from the role of the microbiome in health and disease (Bi *et al.* 2022, Cianci *et al.* 2022, Azevedo *et al.* 2023) to ecosystem dynamics (Follows *et al.* 2007, Freilich *et al.* 2010). Microbial abundance patterns are sparse matrices containing abundances of taxa either as a time series of a particular sample or for various samples. A rich ecosystem of network inference algorithms (Faust *et al.* 2012, Fang *et al.* 2015, Li *et al.* 2023) has evolved around this data type, along with careful discussions of challenges and artefacts (Faust 2021) in the identification of interaction networks.

Based on accumulating evidence (Giliberti *et al.* 2022, Zhu *et al.* 2022) that important aspects of a microbial system can be explained by the presence and absence of microbial taxa and that this is a relevant level of information (and distinct from the information content of the sizes, i.e. the actual abundances, themselves), we binarize the abundance patterns. Binarizing the abundance patterns also dismisses the bias from the highly abundant taxa. Furthermore, focusing on binarized abundance patterns allows us to use a fundamentally different mathematical language for the interpretation, namely Boolean dynamics, and the notion of attractors in Boolean networks [first introduced in Kauffman (1969)]. Attractors in Boolean networks are a set of states of nodes in a network that represent the long-term behaviour of the system. For each initial state of a node, its future state is determined by specific logical rules [see Equation (1)] applied to the state of their neighbouring nodes. By considering binarized abundance patterns, one assumes that the important biological states can still be described by these Boolean states.

In a different application domain, gene regulatory networks (GRNs), the challenge of Boolean network inference is well-known. Numerous strategies of Boolean network inference have been developed and studied (Trinh and Kwon 2021, Beneš *et al.* 2023). The fundamental (algorithmic) difference to microbial interaction networks is the type of data entering the inference problem: While microbial interaction networks are inferred from abundance patterns, inference of GRNs is typically based on gene knockout data (and, often, additional constraints derived from literature) (Chevalier *et al.* 2019, Beneš *et al.* 2023). Even though various computational methods exist for community modelling to study the nature of microbes within a community (Heirendt *et al.* 2019, Diener *et al.* 2020, Blasco *et al.* 2024), this level of comprehensive experimental detail is not yet available for microbial abundance data.

Here, we use the hypothesis that binarized abundance patterns are the attractors of the underlying microbial interaction network. We start from a previous attempt to operationalize this attractor concept, the ESABO (Claussen *et al.* 2017) method. This self-consistent ESABO attempts to infer the significant synergistic and competitive links between pairs of microbial taxa by quantifying their co-occurrences under a null hypothesis, and to create a network of microbial interactions. Here, we use an analytical approach to ESABO [as described in Mendler *et al.* (2024)].

ESABO essentially estimates positive and negative edges from the patterns of co-occurrences of Boolean states. A more apt algorithmic task to go beyond ESABO would be: Is the network capable of reproducing (only) the observed (binarized) abundance patterns as attractors under a simple majority-vote update rule (reliable prediction). Even though, for simulated data, ESABO gives a fair network prediction, there is often a low overlap between the attractors generated by the predicted network and the original network. Even for small networks, the search space is too vast to merely screen the space of all possible networks satisfying a set of attractors. Recently, a version of ESABO enhanced by simulated evolution has been formulated (Mendler *et al.* 2024). This method uses ESABO as a ‘head start’ in the search (via simulated annealing) of a network, which has the full set of available (binary) abundance patterns as attractors. The method has the drawback of high computational demand but can generate reliable networks.

In this work, we address the same question by using a more ‘microscopic’ approach, studying the change in the behaviour of attractors as a consequence of edge perturbations in the network. On this principle, we designed an algorithm that extracts information from the comparison of attractor sets of two networks (original and partially predicted by ESABO), to determine the differences between the respective networks. Using this information iteratively, we make changes in the ESABO network to bring it closer to the original network by narrowing the gap between the attractor sets generated by the two networks.

We first study the algorithm on simulated data for signed (edges labelled with +, −), undirected networks. We consider small-scale networks to be able to exhaustively enumerate the attractors of the system. Hence, the application to microbial networks is on the phylum level. Application at lower (and probably more informative) taxonomic levels requires the discussion of incomplete attractor sets, which we briefly touch upon in the discussion.

## 2 Materials and methods

### 2.1 Boolean threshold model and attractor set computation

Consider an undirected signed random network G=(μ,E) with node set μ and edge set E. The network has N=|μ| nodes and $M_{+}$ positive and $M_{-}$ negative undirected edges, $M_{+}+M_{-}=|E|$. From this network, we can generate an interaction matrix I, with $I_{ij}=+1$, −1 and 0 for positive, negative and no interaction between the nodes i and j, respectively. In the context of microbial communities, this graph G can be thought of as a microbial interaction network summarizing the positive (synergistic) and negative (competition) interactions among the N microorganisms. Binary dynamics (simulating presences, $s_{i}(t)=1$, and absences, $s_{i}(t)=0$, of microorganism i at time t with i=1,2,…,N) can now be simulated via a simple threshold type (or ‘majority vote’) update rule (see Li *et al.* 2004, Szejka *et al.* 2008):


(1) $$s_{i}(t+1)=\{\begin{array}{cc} 1, & \sum_{j=1}^{N}\;I_{ij}s_{j}(t)>0\\ 0, & \sum_{j=1}^{N}\;I_{ij}s_{j}(t)<0\\ s_{i}(t), & \sum_{j=1}^{N}\;I_{ij}s_{j}(t)=0 \end{array}$$

for the state $s_{i}(t+1)$ at node i at time t+1. Updating each of the $2^{N}$ possible initial states exactly once using the update rule, Equation (1), yields a directed graph with system states as nodes and edges s(t)→s(t+1). The nodes with zero out-degree (fixed-point attractors) and the cycles (cyclic attractors) in this graph form the *attractor set* of the system. With the connectivity and network sizes studied here, the vast majority are fixed-point attractors.

### 2.2 Attractor-based network change predictor

**Figure 1.**
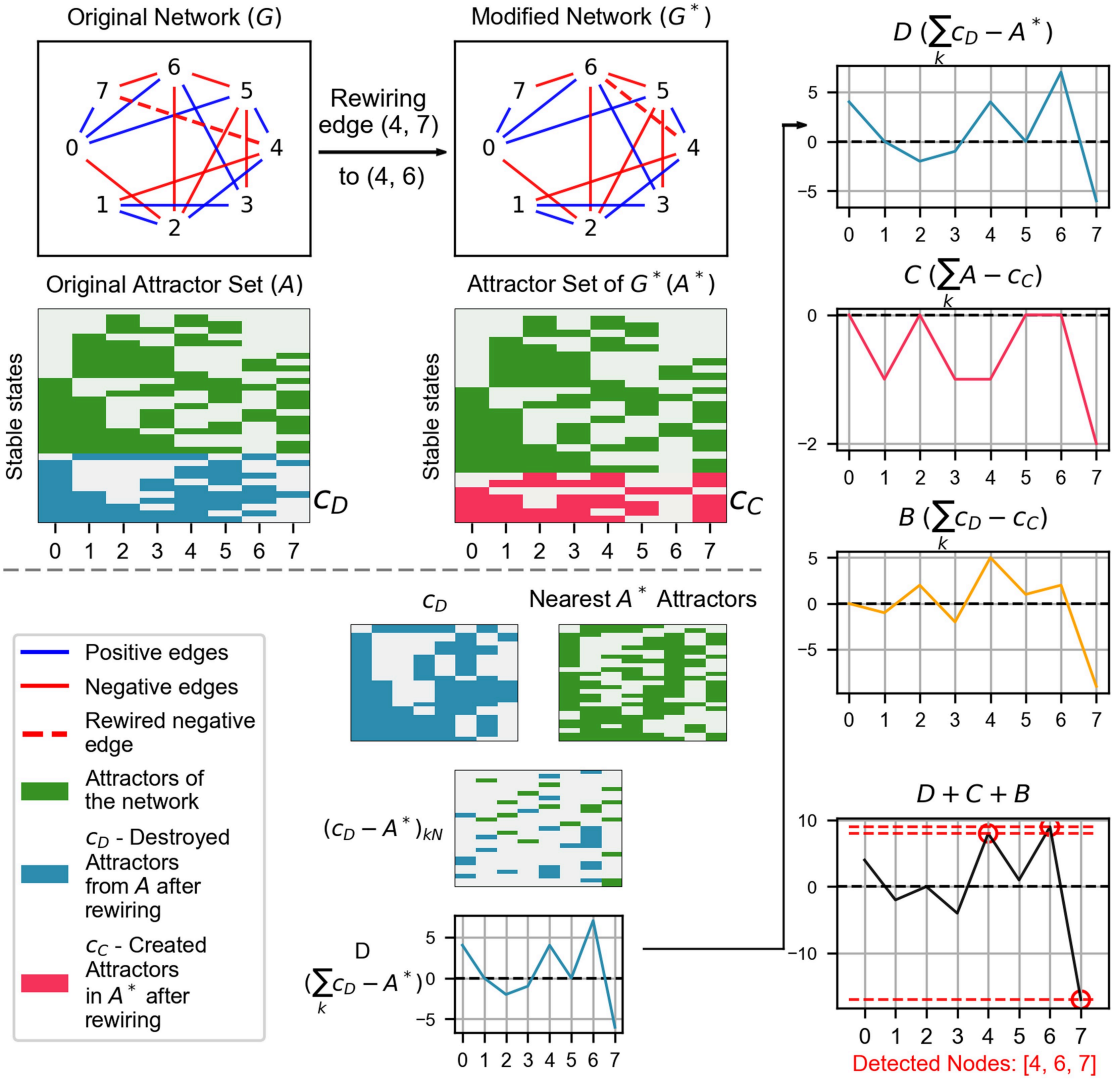
Schematic representation of the process of modified edge detection. Edge (4,7) in *G* is rewired to edge (4,6) in $G^{*}$. Boolean attractors of the respective networks, obtained by using update rule in Equation (1), are *A* and $A^{*}$, with light colours representing 0 s and dark colours, 1 s. In the bottom section, ${\left(c_{D}-A^{*}\right)}_{kN}$ is found by element-wise subtraction of $c_{D}$ and its nearest $A^{*}$ attractors. It consists of 0, 1, and −1. On summing over *k*, we obtain *D*, as shown at the bottom of the middle column. Similar comparisons of attractor sets (see Section 2) yield the vectors depicted in the rightmost column: In addition to *D*, the arrays *C*, *B* are shown. As the last entry in this column, summation of the three arrays gives a final array with the highest peaks including the rewired nodes.

In order to study the sensitivity of attractors to small changes in G we rewire a single edge $e_{1}=(n_{0},n_{-})$ to $e_{1}^{*}=(n_{0},n_{+})$, where $n_{-}$ denotes a node that lost an edge, $n_{+}$ denotes the node that gained an edge and $n_{0}$ denotes the anchor node of the rewired edge. Here $e_{1}\in E$ and $e_{1}^{*}\notin E$. The modified network generated after rewiring an edge is denoted by $G^{*}$. Let the attractor set of the original network (G) be denoted by A, and of the modified network ($G^{*}$) be denoted by $A^{*}$. Let $c_{D}$ denote the complement $c(A,A^{*})=A\backslash A^{*}$, i.e. destroyed attractors and $c_{C}$ denote the complement $c(A^{*},A)=A^{*}\backslash A$, i.e. created attractors. These sets are illustrated in Fig. 1.

#### 2.2.1 Comparing attractor sets of two networks to identify nodes highlighting their differences

As a first component of an algorithm to identify the edge change in networks G and $G^{*}$, we analyse various aspects at node-level changes in the attractor sets for G and $G^{*}$ (Fig. 1). For each attractor $a_{i}^{D}$ in $c_{D}$, we find its nearest attractors, $a_{k(i)}^{*}$, in $A^{*}$ by least Hamming distance [see definition in Supplementary Information (Supplementary Appendix S1)]. For each $a_{k(i)}^{*}$, we obtain an element-wise subtraction $a_{i}^{D}-a_{k(i)}^{*}$, to get a collection of k numeric arrays of length N containing 1,0,−1, $\left(d_{1},d_{2},\ldots ..,d_{k}\right)$. Then, we stack these arrays on top of each other to get a matrix ${\left(c_{D}-A^{*}\right)}_{\left(kN\right)}$. Summing over the rows of this matrix gives the final summed array $\sum_{k}c_{D}-A^{*}=D$ (blue curve in Fig. 1). Similarly, we obtain $\sum_{k}A-c_{C}$ by finding, for each attractor in $c_{C}\left(a_{i}^{C}\right)$, its nearest attractors in $A\left(a_{k(i)}\right)$. Then, element-wise subtraction $a_{k(i)}-a_{i}^{C}$ gives a collection of arrays, $\left(c_{1},c_{2},\ldots ..,c_{k}\right)$, stacked as matrix ${\left(A-c_{C}\right)}_{\left(kN\right)}$, which summed over k rows generate an array $C=\sum_{k}A-c_{C}$ (pink curve in Fig. 1). Lastly, for $c_{D}$, we find nearest attractors in $c_{C}$, and generate summed array B = $\sum_{k}c_{D}-c_{C}$ (yellow curve in Fig. 1). D+C+B gives a final array (black curve in Fig. 1) whose highest and lowest values reveal the nodes that were involved in the rewired edge. Figure 1 shows a worked out example.

**Figure 2.**
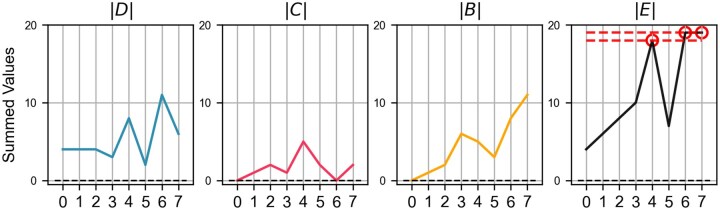
The arrays (*D*, *C*, *B*) shown in Fig. 1 were generated by summing across rows derived from differences between the close attractors of the two attractor sets (see Section 2.2.1 for details). Here, we compute the absolute values of each array before summing them to produce the absolute summed arrays. The resulting $S_{N}$ is [6,7,4,3,2,5,1,0].

For convenience, as well as to not lose information due to adding positive and negative values in matrices, we take the absolute values of the arrays in ${\left(c_{D}-A^{*}\right)}_{\left(kN\right)}$, i.e. $\left(|d_{1}|,|d_{2}|,\ldots ..,|d_{k}|\right)$, and then sum over $k,\sum_{k}|c_{D}-A^{*}|=|D|$. Similarly, we obtain $\sum_{k}|A-c_{C}|=|C|$ and $\sum_{k}|c_{D}-c_{C}|=|B|$, then $\sum_{k}|c_{D}-A^{*}|+\sum_{k}|A-c_{C}|+\sum_{k}|c_{D}-c_{C}|$ is denoted by |E| (Fig. 2). We associate each element in |*E*| to its node, such that each element contains the information → (node: peak height). If |*E*| is sorted with respect to the height of the peaks (descending), we get a sorted array $S_{N}$ (sorted nodes), with nodes involved in the rewiring at the initial positions (see Supplementary Fig. S1 in Supplementary Information).

#### 2.2.2 Method calibration: predicting networks with multiple rewired edges

Next, we attempt to predict more than one rewired edge in a network. The algorithm (python pseudo-code) is given in Supplementary Appendix S2 in Supplementary Information.

**Procedure:** In order to calibrate the method, a network G is generated with N nodes, $M_{+}$ positive and $M_{-}$ negative undirected edges. Then, a modified network, $G^{*}$, is created by randomly rewiring n edges in G. Rewiring is done in a way such that $G^{*}$ is a connected network and no multiple edges are created. The attractor set of G is A, and the attractor set of $G^{*}$ is $A^{*}$.

**Algorithm (EDAME: *Edge detection via attractor mismatch evaluation*) objective**: Compare the attractors A and $A^{*}$ to identify the differences at the node level. The findings of this comparison will then be used to evolve the network $G^{*}$ into a network that can produce the attractor set A. It is noteworthy that this network frequently results in G.

In this algorithm, we take $G^{*}$, $A^{*}$, A and $I^{*}$ (Interaction matrix of $G^{*}$) as inputs. We define a list of possible combinations ($P_{C}$) of states that a pair of edges can have, e.g. (−1, 0) implies that the first edge in the pair is a negative edge, and the second one is not an existing edge. Then, we find the overlap between A and $A^{*}$, $JI_{A}$ (Formula in Supplementary Appendix S1 in Supplementary Information), followed by finding $S_{N}$ (sorted nodes) for A and $A^{*}$, as described in Section 2.2.1. In $S_{N}$ vector, we choose the first 2 nodes and combine them with the 3rd, 4th, 5th, and so on nodes, iteratively, to obtain a list of 3 nodes ($N_{\mathrm{list}}$) each time. Then, we make combinations of two nodes from $N_{\mathrm{list}}$ to get a list of edges ($E_{\mathrm{list}}$). Again, creating combinations of two edges in this list gives a list of edge pairs ($E_{\mathrm{pair}}$), which are then iteratively checked by changing the state of an edge pair to all possible combinations except its own combination, to create a temporary network $G_{t}$ with an attractor set $A_{t}$.

- If the overlap between A and $A_{t}$ ($JI_{t}$) is lower than the overlap between A and $A^{*}$ ($JI_{A}$), then we continue checking other possible combinations, if none of the combinations give an increase in the overlap, we move to the next $N_{\mathrm{list}}$. If none of the node lists of size three give $JI_{t}>JI_{A}$, then we move to node lists of size four, five and so on until N.
- If $JI_{t}>JI_{A}$ and $JI_{t}\neq 1$, then we accept the edge change and assign $G_{t}$ as $G^{*}$ and $A_{t}$ as $A^{*}$, and start from the beginning with finding out $S_{N}$ again.
- Lastly, if $JI_{t}=1$, then we break all the loops and accept $G_{t}$ as $G^{*}$ and finally as $G_{\mathrm{end}}$. This is the predicted network.

In some cases, we exhaust all the node lists and $JI_{t}$ still has not reached 1, then we call this a failure of the algorithm and start the whole process again with randomized edge pairs (explained in Section 3.2).

## 3 Results

**Figure 3.**
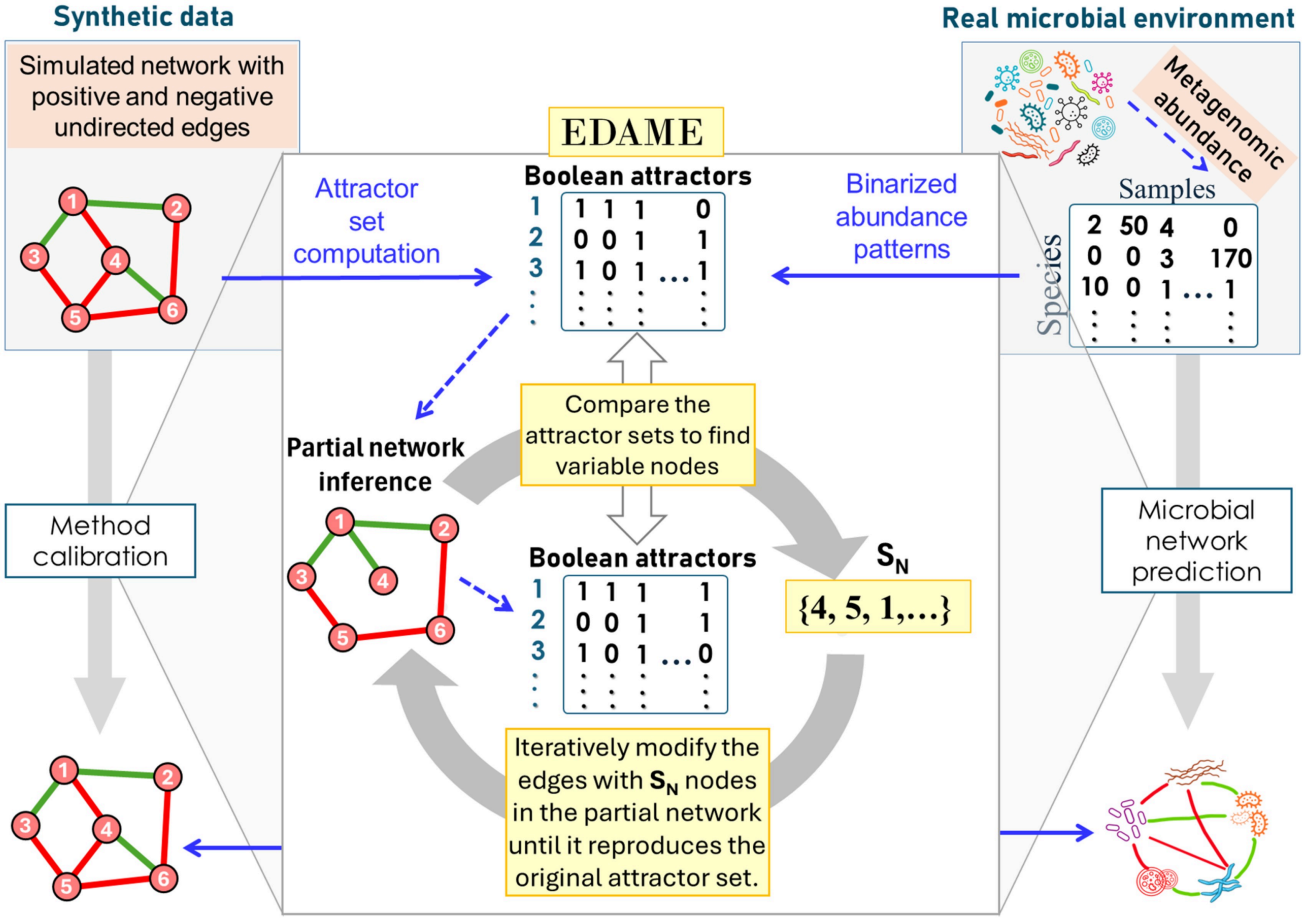
Structure of the study. The study consists of two main components: (i) Method Calibration, performed using simulated data generated from a synthetic network (left column), and (ii) Microbial Network Prediction, conducted on real metagenomic abundance data (right column). Boolean attractors are derived from both data types, and the EDAME algorithm is applied to infer the original network structure of the system.

As illustrated in Fig. 3, the study design is structured into two main phases: (i) Method calibration and (ii) Microbial network prediction. The calibration of the *EDAME* method involves the generation of synthetic abundance data from a simulated undirected signed network, and using the ‘majority vote’ update rules [see Equation (1)], Boolean attractors are subsequently derived from this network. These Boolean attractors then serve as the basis for inferring the simulated network via the ESABO method (Claussen *et al.* 2017). The inferred network represents only a partial reconstruction, as the attractors generated from the ESABO network deviate from those of the original simulated network. By comparing these attractor sets, we identify variable nodes, referred to as ‘sorted nodes’ ($S_{N}$) (see Section 2.2.1 and SI Text), along with their associated edges. Through an iterative refinement process, we systematically adjust the edges of the partially inferred network until the resulting attractors align with those of the original simulated network (see Fig. 3 as an example). Section 3.2 provides an evaluation of the performance of the EDAME algorithm on simulated data and a comparison of its effectiveness against other network inference tools.

In the second phase of the study, the EDAME algorithm is applied to real microbial abundance patterns (see Section 3.4). In this context, metagenomic abundance patterns are binarized and interpreted as the Boolean attractors of the underlying microbial interaction network. The EDAME algorithm is subsequently applied to these Boolean attractors to infer the microbial interaction network. The effectiveness of the EDAME algorithm relies on the ability of the ‘sorted nodes’ array ($S_{N}$) to detect changes in the edges of the network, which is derived from the systematic perturbation of attractors induced by edge modifications. A detailed performance assessment of $S_{N}$ is provided in the Supplementary Information (SI Text).

### 3.1 Enhancement of initial networks by EDAME

Here we illustrate with an example how sensitivity of attractors with respect to small changes in the network can be utilized to predict a network from its partially predicted network version (see Section 2 for the underlying algorithm). In an iterative application of the algorithm EDAME, we evolve a partially predicted network to a network that has the same set of attractors as those of the original network.

**Figure 4.**
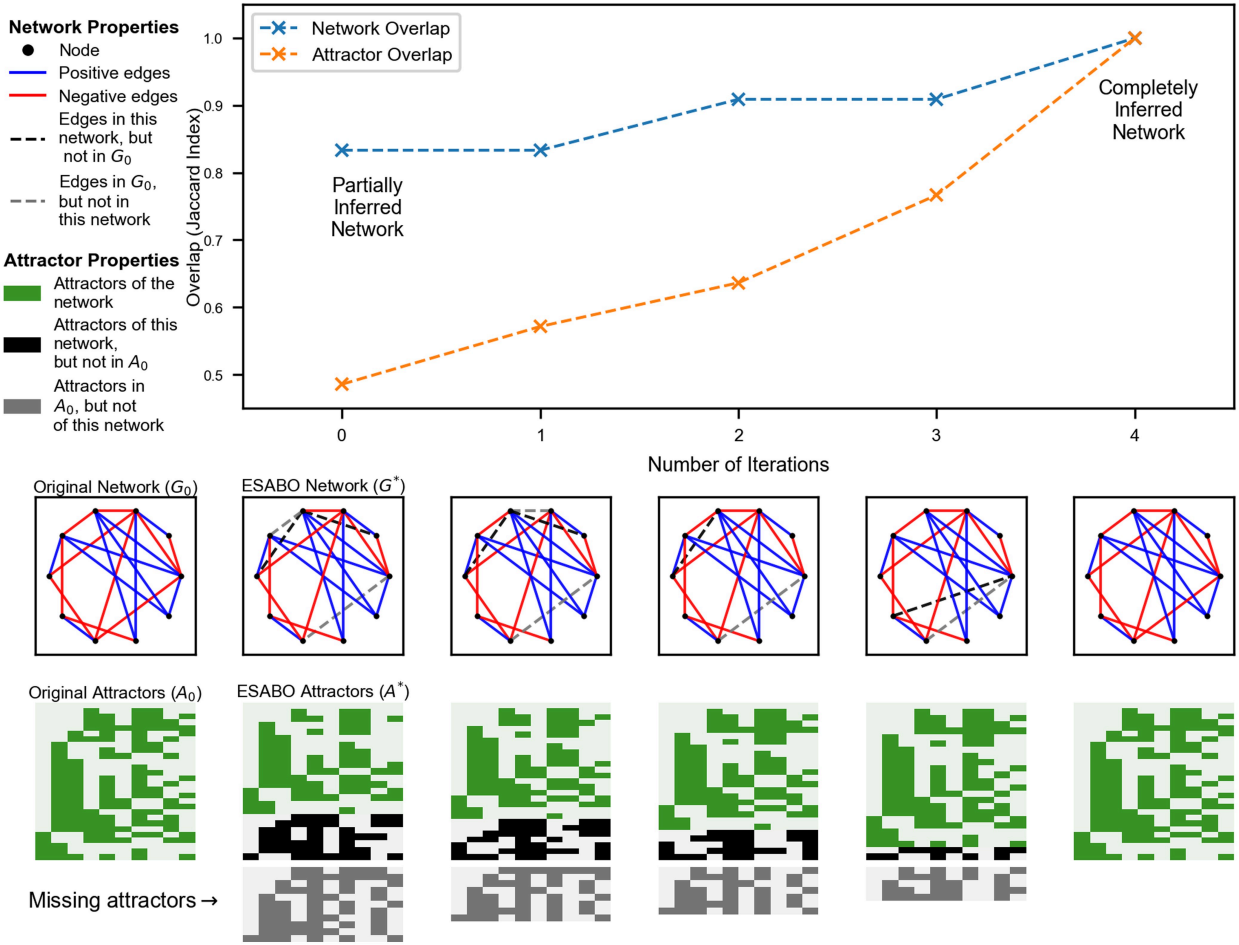
Evolution of partially inferred ESABO network, $G^{*}$, to a network with the same attractor set as $A_{0}$, which gives us the original network, $G_{0}$. Network parameters are $N=M_{+}=M_{-}=10$. Shown below the networks are the attractors of the networks with green: attractors matching to $A_{0}$; black: attractors absent in $A_{0}$; grey: attractors present in $A_{0}$ but missing in the corresponding attractor set. The algorithm takes four iterations to correct four edges (two false positives, two false negatives), shown as dashed edges in the network $G^{*}$.

We simulate a random undirected network $G_{0}$ with N nodes, $M_{+}$ positive edges, $M_{-}$ negative edges, then find attractors of the network, $A_{0}$, using synchronous update rules given in Equation (1). The initial network is inferred using the ESABO (Claussen *et al.* 2017, Mendler *et al.* 2024) technique. ESABO (*Entropy Shifts of Abundance Vectors under Boolean Operations*) is a network inference method that uses the co-occurrence of two nodes in the attractors to calculate entropy shift scores assigned to each possible edge in the network. Edges of the network are deduced based on these scores. Edges with a score above a certain positive threshold are positive edges and below a negative threshold are considered negative edges in the inferred network. The network inferred by ESABO is denoted by $G^{*}$, with attractor set $A^{*}$. In most cases, ESABO is only able to predict the network partially, hence $G^{*}$ is not equal to $G_{0}$. We apply EDAME on $G^{*}$ to reach $G_{0}$, as shown in Fig. 4. Even though the initial network overlap at 0th iteration is above 80%, attractor overlap is at 50%. This illustrates that small changes in networks can lead to large changes in attractor sets. Implementation of the EDAME algorithm with ESABO on a simulated network is given at https://github.com/Jojo6297/edame.git.

### 3.2 Evaluation of the EDAME algorithm

**Figure 5.**
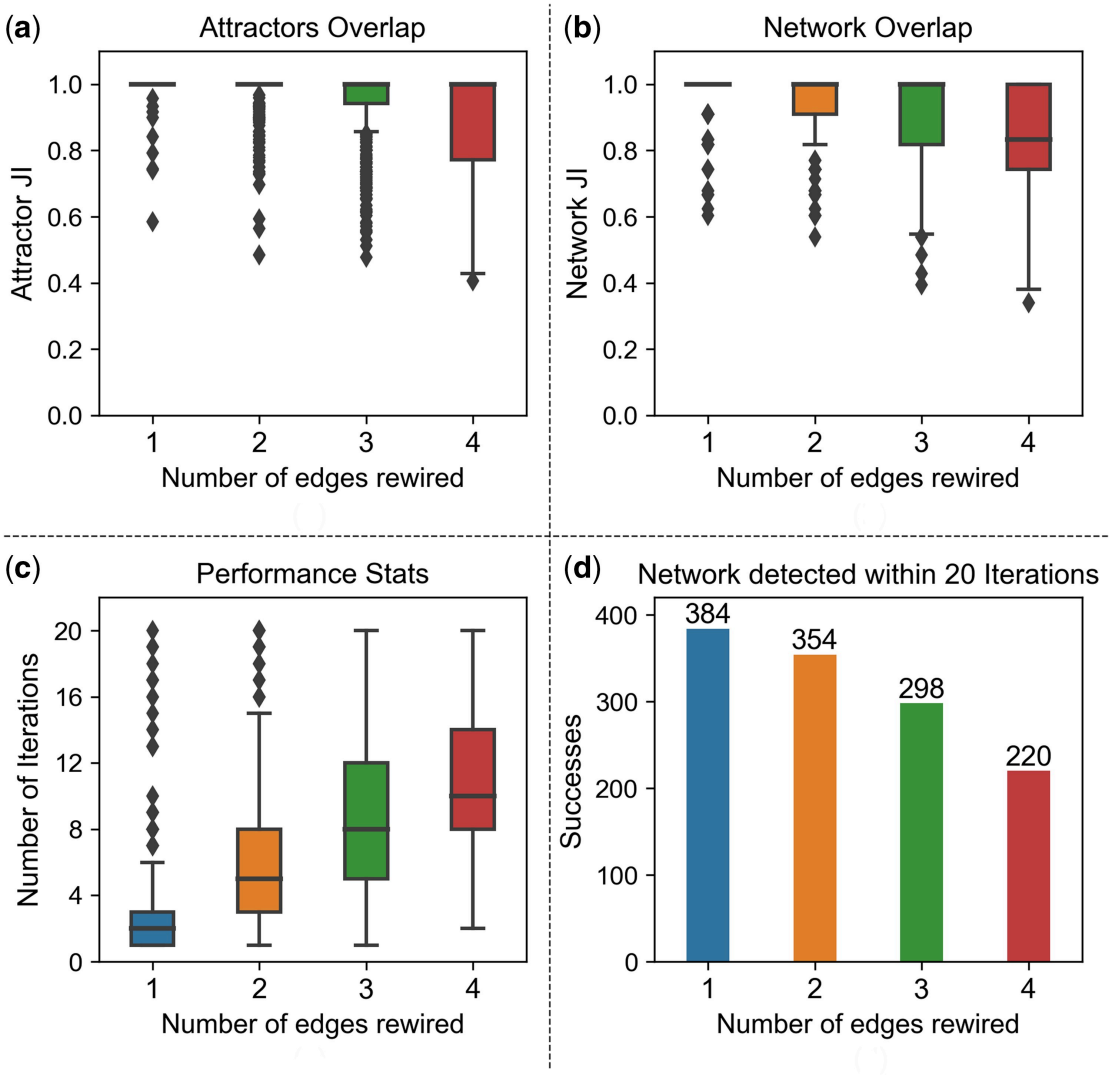
(a) Box plot of the overlap of attractor sets *A* and $A^{*}$ at the end of the algorithm. Overlap =1 implies the algorithm was successful, failed otherwise. (b) Overlap of networks *G* and $G_{\mathrm{end}}$. (c) Box plot of the number of edge iterations the algorithm underwent to succeed (failures excluded). (d) Number of successful predictions out of 400 experiments. Note that one rewired edge introduces one false positive and one false negative in the modified network, therefore one rewired edge equals two edge differences between *G* and $G^{*}$. Iteration and failure limits of $l_{it}=20$ and $l_{fa}=20$ have been used.

Next, we measure the performance of the algorithm on simulated networks. In Fig. 5, we show a collective statistical result from 400 independent network prediction experiments, for the number of rewired edges ∈{1,2,3,4}, with upper limits imposed on the number of iterations and the number of failures of the algorithm. The algorithm terminates in one of three possible ways: (a) The algorithm is able to find and correct all rewired edges. (b) A network that produces the same set of attractors as the original network, but is not equal to the original network, is found. These networks are mostly very close to the original network. (c) The algorithm was not able to find a network that can reproduce the same attractor set. We consider scenario (a) and (b) as a success and (c) as a failure.

With upper limits on the number of iterations and failures, Fig. 5 shows, as expected, that the performance of the algorithm decreases with increasing number of rewired edges. The algorithm performs better, if the network to be predicted is not too distinct from the network at hand. With increasing edge differences, the topological information starts to share nodes in the attractor differences, hence, the signals are not entirely reliable. Therefore, the algorithm corrects for edges sequentially and calculates $S_{N}$ after each iteration to reduce noise in the topological information.

More statistical results comparing the attractor overlap, network overlap, failed attempts and success rate with respect to the difference in edges are given in the Supplementary Information (Supplementary Figs S3 and S4).

### 3.3 Comparative analysis of EDAME with network inference algorithms

**Figure 6.**
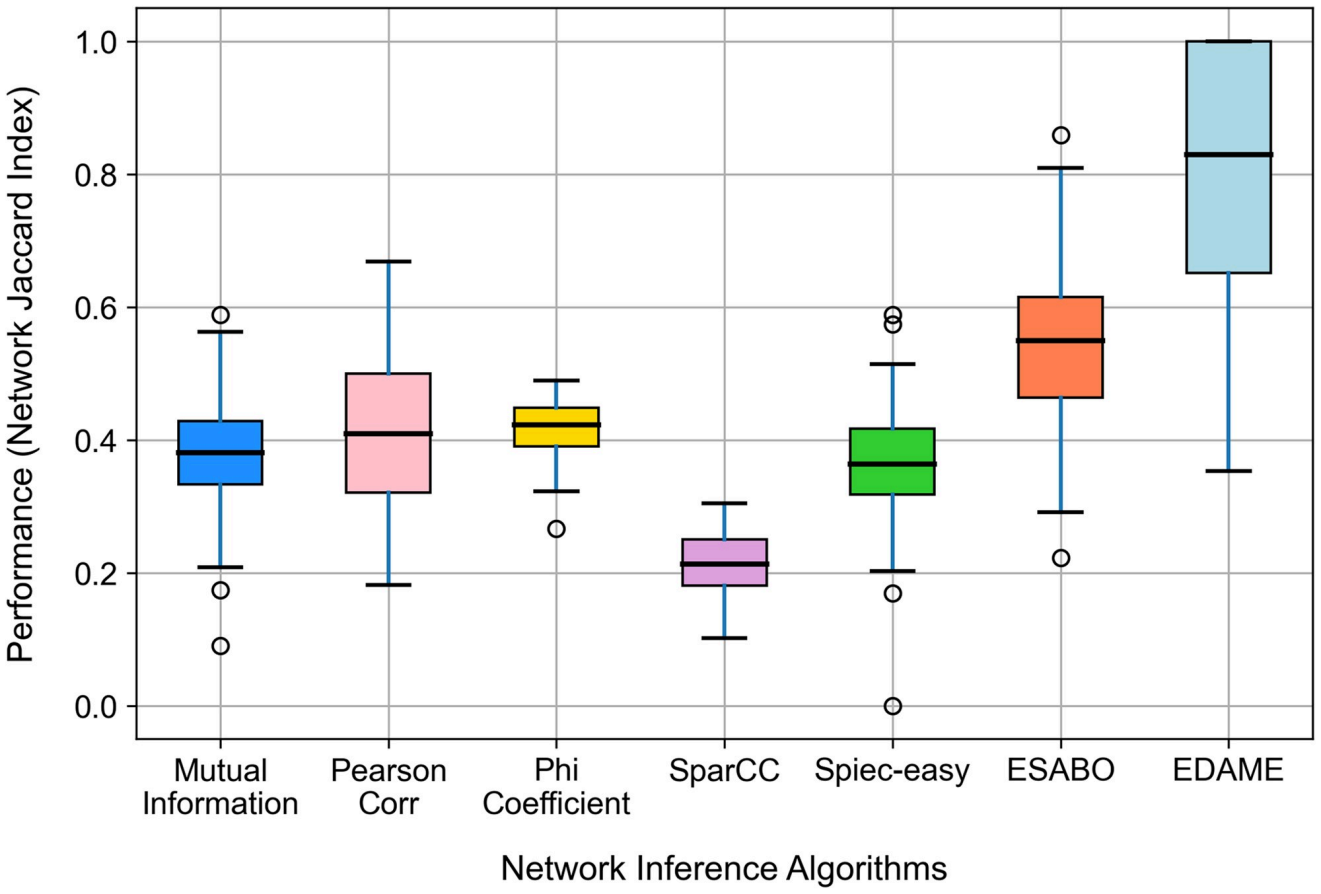
Performance comparison of various network inference methods. The accuracy of each method is evaluated based on its ability to reconstruct the original network from 100 synthetic abundance datasets.

In order to evaluate the performance of the EDAME algorithm in comparison to existing network inference methods, 100 random undirected networks were generated using the following parameters: N=10, $M_{+}=10$, and $M_{-}=10$. Subsequently, Boolean attractors were then generated from these networks, and a variety of network inference algorithms were applied to each attractor set (Fig. 6) in order to determine the success rate of each method. The evaluated approaches include general network inference techniques such as mutual information, Pearson correlation, and the phi coefficient, as well as microbial network-specific methods, including SparCC (Friedman and Alm 2012), SPIEC-EASY (Kurtz *et al.* 2015), and the ESABO inference method.

As demonstrated in Fig. 6, the EDAME algorithm demonstrates superior performance in comparison to alternative methods, attaining a median Jaccard Index (JI) of approximately 0.82 across all inferred networks. The ESABO method exhibits a median JI of approximately 0.53, while the remaining methods predominantly yield JI values below 0.5.

However, caution should be exercised when comparing SparCC and SPIEC-EASY with the other methods, as these approaches are not specifically designed for binary data. In order to ensure compatibility with SparCC and SPIEC-EASI, we transform Boolean attractors into abundance patterns that match the required data format. For each network, we compute attractors for all possible initial conditions ($2^{10}=1024$). We then randomly select 200 attractors, sum their values, and repeat this process 1024 times, yielding 1024 integer-valued samples. From these, we extract only the values corresponding to 1 s in the network-derived attractors. These processed samples serve as abundance patterns for constructing networks using SparCC and SPIEC-EASI. While this approach does not enable an equitable comparison, it does facilitate a relative comparison. Additionally, an alternative method was considered, which used a log-normal distribution to transform Boolean attractors into abundance patterns. However, the approach described above produced superior results for SparCC and SPIEC-EASI, and therefore it was adopted for the analysis.

### 3.4 Applications to human microbial data

In the sections above, we presented results for simulated data. Here, we apply the algorithm in combination with ESABO to human microbial abundance data to estimate microbial interaction networks. There are various data resources (Peterson *et al.* 2009, Mitchell *et al.* 2020) that provide microbial abundance patterns for a range of conditions. We use a Bioconductor package in R, curatedMetagenomicData (Pasolli *et al.* 2017), to obtain standardized, curated human microbiome abundance patterns. For a small meta study, we chose 16 studies (4 studies each) with stool samples belonging to healthy human subjects (Zeevi *et al.* 2015, Zhernakova *et al.* 2016, Mehta *et al.* 2018, Asnicar *et al.* 2021), human subjects with type 2 diabetes (T2D) (Qin *et al.* 2012, Karlsson *et al.* 2013, Li *et al.* 2014, Ho *et al.* 2020), human subjects with colorectal cancer (CRC) (Zeller *et al.* 2014, Vogtmann *et al.* 2016, Wirbel *et al.* 2019, Yachida *et al.* 2019), and human subjects with inflammatory bowel disease (IBD) (Nielsen *et al.* 2014, Hall *et al.* 2017, Schirmer *et al.* 2018, Vich Vila *et al.* 2018, Lloyd-Price *et al.* 2019). For each study, we compute three types of networks:

- Spearman’s correlation (*p*-value <0.05) with varying thresholds (0.1, 0.2, 0.3, 0.4).
- ESABO network with varying edge selection (number of positive edges = {4, 5, 6, random}, number of negative edges = {2, random})
- EDAME network with varying ESABO networks as initial states

The Correlation analysis is done on non-binarized data and to obtain Boolean attractors for ESABO and EDAME analysis, we extract the normalized abundance patterns for each study, sum over the populations of species to the phylum level, binarize the counts and consider the unique abundance vectors as attractors of the system. We restrict the analysis to the intersection of microbial species from the 16 studies to be able to compare output networks. We run *ESABO* + *EDAME* on the attractors with $l_{fa}$ (failure limit) = 50. Out of 50 runs, we choose the experiment which gives the maximum Jaccard index between the original attractors and the attractors obtained by the predicted network before failing.

Following the steps above, we obtain 16 (4 healthy, 4 T2D, 4 CRC, 4 IBD) networks for each network inference tool (Correlation, ESABO, EDAME) for all varying initial conditions and thresholds. Given that the networks that belong to the same cohort should be more similar to each other than to networks that belong to different cohorts, we create a quality index (QI) to compare the three network inference methods, i.e. ratio of similarity of networks belonging to the same cohort to the similarity of networks across different cohorts. The abundance patterns of the four studies reveal that T2D and CRC acts as a control between the healthy and IBD states (see Supplementary Fig. S5 in Supplementary Information). The overlap of abundance patterns of T2D and CRC with healthy samples is much higher than IBD samples. Therefore, we take T2D and CRC out of the normalization and consider only the healthy and IBD cohorts for calculating the QI. The formula for QI is given in Supplementary Appendix S1 in Supplementary Information.

**Figure 7.**
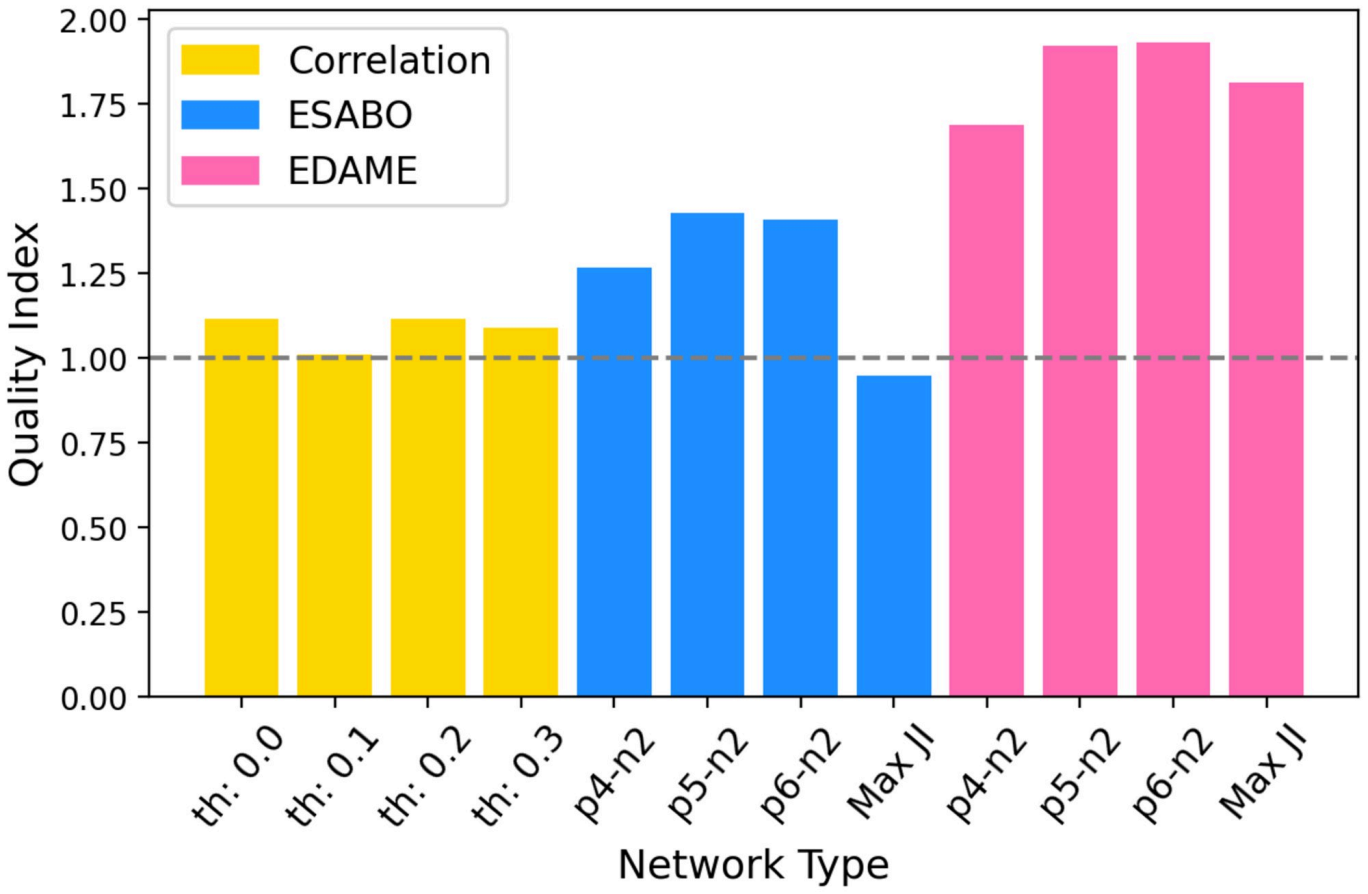
Comparison of quality index of the three network inference tools: Correlation networks (with thresholds = (0,0.1,0.2,0.3)), ESABO networks (with the edge selection: positive edges = (4,5,6,random), negative edges = (2,random)), EDAME networks (with the respective ESABO networks as initial networks.).

The comparison (Fig. 7) reveals that the two methods based on binary data (ESABO and EDAME) segregate the two health conditions (healthy/IBD) much better than correlation networks. Within the two Boolean methods, EDAME leads to a visible increase in this quality indicator, compared to ESABO. The QI-plot including T2D and CRC is shown in the Supplementary Information (Supplementary Fig. S6). In this case as well, ESABO and EDAME segregate the cohorts better than correlation networks but with a much weaker signal.

**Figure 8.**
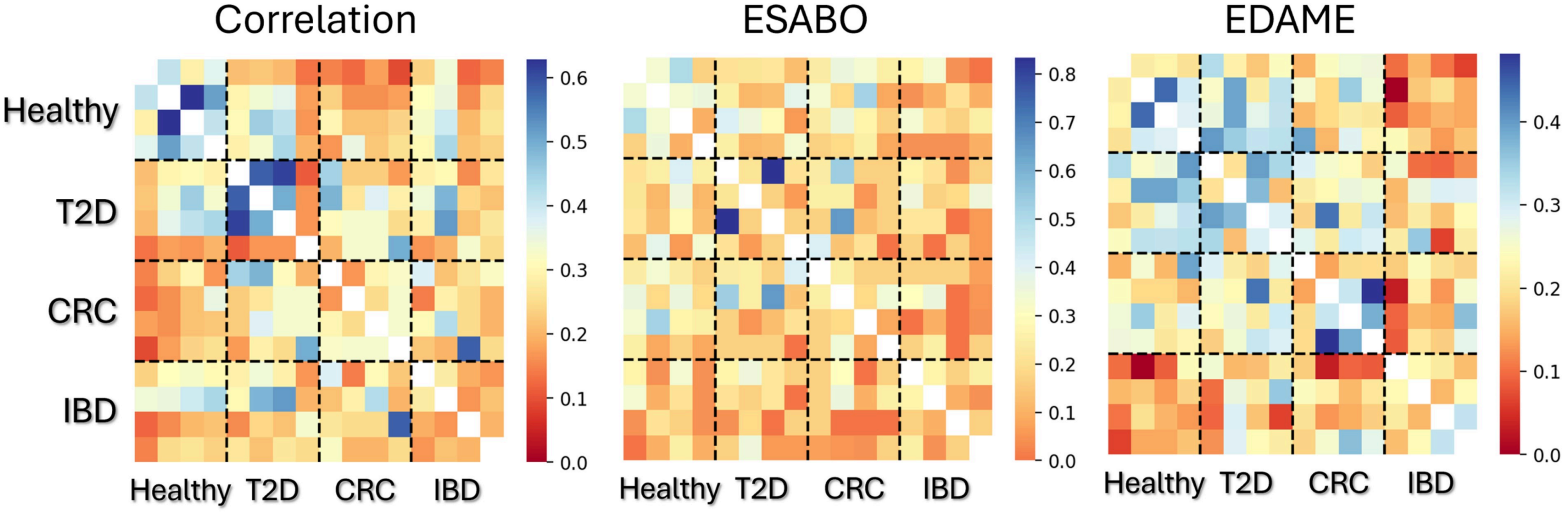
Heatmaps showing the Jaccard similarity among the networks obtained by correlation, ESABO, EDAME for the four cohorts: healthy, T2D, CRC, IBD.

Figure 8 shows the Jaccard similarity among all the networks for the three network inference techniques. EDAME algorithm shows the highest segregation of healthy, T2D and CRC networks from IBD. See comparison of various initial conditions in Supporting Information (Supplementary Fig. S7).

To underscore the plausibility of disease segregation as identified via EDAME, we conducted a literature analysis using a brute-force approach in order to assess the relevance of the gut microbiome across a range of diseases. Specifically, publications from 2020 to 2024 containing the terms ‘gut microbiome’ + ‘< disease>’ were queried in Google Scholar and the results were normalized by the total number of publications for ‘<disease>’ alone. For instance, a search for ‘gut microbiome’ ‘Inflammatory Bowel Disease’ yielded 34 600 results, whereas ‘Inflammatory Bowel Disease’ alone returned 108 000, indicating that approximately 32% (34 600/108 000) of IBD-related publications also reference the gut microbiome. Conversely, the gut microbiome is mentioned in 8.66% of colorectal cancer (CRC) publications and 5.52% of diabetes-related studies. This percentage is interpreted as a broad indicator of dysbiosis in each disease state. While T2D and CRC have relatively fewer publications discussing the gut microbiome, IBD-related research exhibits a significantly higher prevalence of microbiome studies. This phenomenon aligns with the pronounced dissimilarity observed between healthy and IBD networks generated by EDAME.

**Figure 9.**
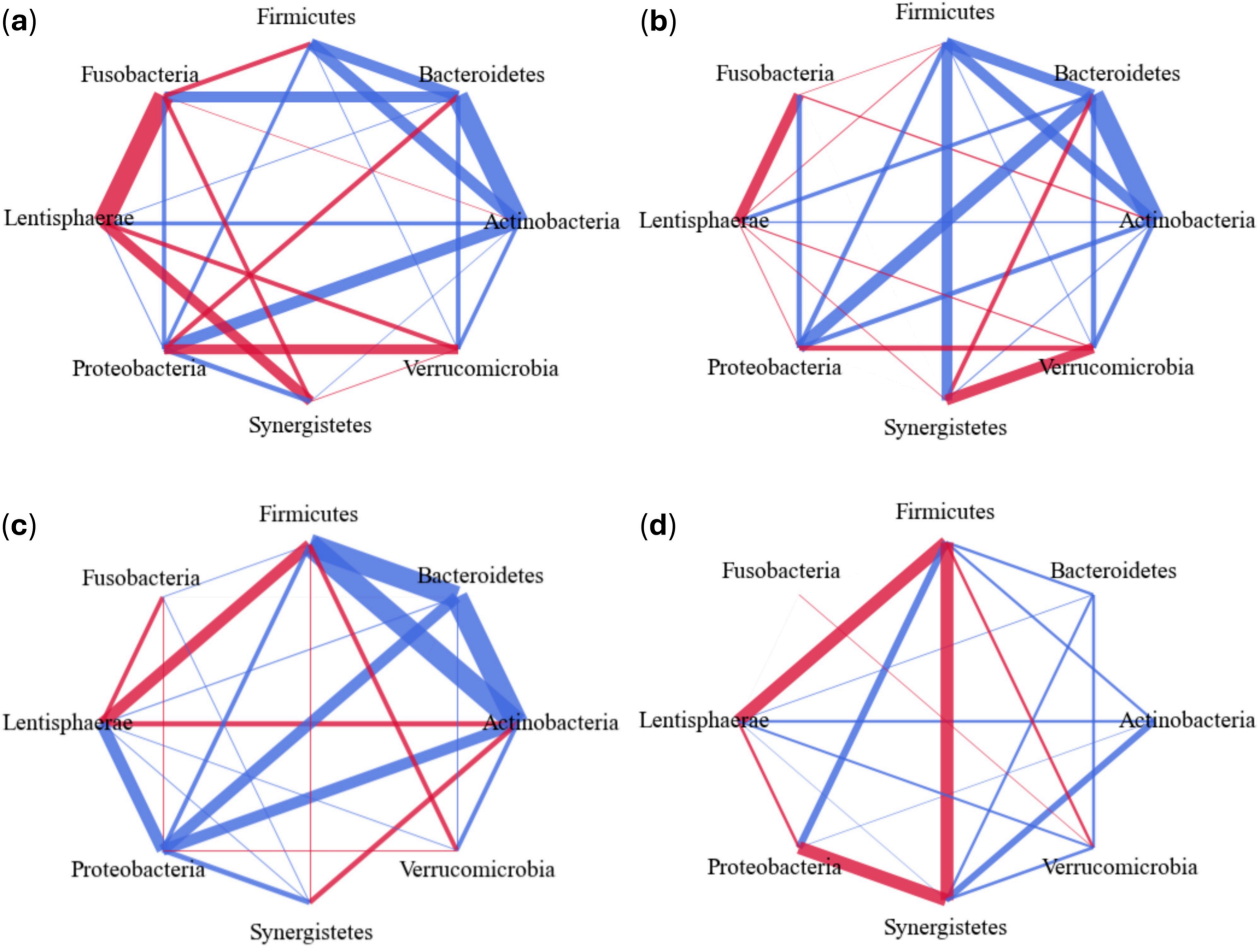
The networks shown are the cumulative EDAME networks generated by overlapping the networks for all four studies for each cohort: (a) Healthy, (b) Type 2 diabetes, (c) Colorectal cancer, and (d) Inflammatory bowel disease. Edge weights are calculated by summing over the edges of four studies multiplied by the attractor JI score at the end of each network prediction.

After the performance assessment of the three algorithms (Figs 7 and 8), we now focus on the EDAME algorithm for an interpretation of the inference results. For each cohort, we generate a summary network by superimposing all the interaction networks inferred from that cohort (Fig. 9). The width of the edges represents the weight of the interaction. The weight of an interaction in the summary network is calculated by summing over that edge (1,0,−1) in each individual network multiplied by the attractor JI score (at the end of EDAME algorithm) of each network.

Figure 9 shows that healthy and T2D interaction networks are denser with higher interaction weights than CRC and IBD, indicating greater consistency across studies. While microbial interactions in T2D, CRC, and healthy states are similar, the CRC network is less consistent than the healthy/T2D networks.

In contrast, IBD networks differ significantly from other states, especially the healthy state. The low intersection among IBD networks across studies results in weaker weighted edges, suggesting high microbial diversity, likely due to disease subtypes or individual variations in microbiome response.

Unfortunately, there is insufficient evidence in literature to compare the network edges with established microbial interactions. Perhaps, the most well-known interaction in the gut is mutualism among the phyla Firmicutes, Bacteroidetes and Actinobacteria (Ríos-Covián *et al.* 2016, Fernandez-Julia *et al.* 2022), which is well conserved in the healthy, T2D and CRC networks shown, but is disrupted in the IBD network with a weak positive edge between Firmicutes and Bacteroidetes and with Actinobateria. In mutiple studies, the F/B (Firmicutes/Bacteroidetes) ratio has been shown to decrease in IBD (Takahashi *et al.* 2016, Stojanov *et al.* 2020). It is interesting to see such clear differences for different health states on such a crude (phylum) level of taxonomy. The healthy and T2D networks seems more stable than the dysbiotic states CRC and IBD networks.

As our EDAME-based analysis is conducted at the phylum level—a broad taxonomic scale—we incorporated a reviewer’s suggestion to perform network inference at the species level based on co-abundance patterns. We then coarse-grained the results to the phylum level and compared them with EDAME networks. However, the inferred networks did not exhibit the same or improved disease segregation observed in our primary analysis. For further details, please refer to the SI text section ‘Inference of microbial phylum networks using co-abundance analysis’.

### 3.5 Inference and comparison of OMM^12^ community interaction networks

Given the limitation of the EDAME algorithm to small communities a good test scenario are minimal model microbiomes, such as the ‘Oligo-Mouse-Microbiota’ (OMM^12^) synthetic bacterial community. In Weiss *et al.* (2022), the authors investigated the interaction dynamics among 12 microbial species within the mouse gut *in vitro*. They constructed a directed interaction network by evaluating strain growth in pairwise co-culture versus monoculture using normalized absolute abundance (see Supplementary Fig. S8a). A significant increase or decrease indicates a positive or negative interaction, respectively, while no significant change (*P* > 0.05) is classified as neutral 0.

**Figure 10.**
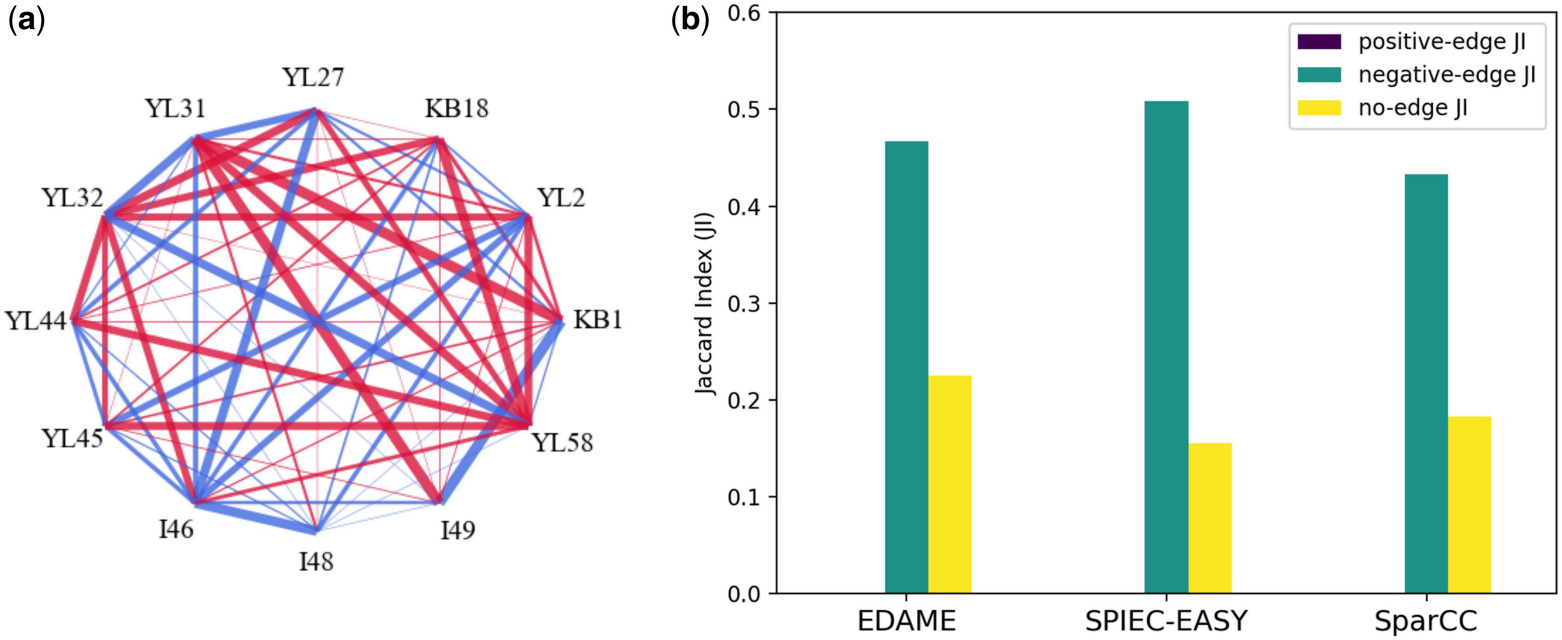
(a) EDAME *in vivo* infant mice network. (b) Comparison of EDAME, SPIEC-EASY and SparCC method with network given in Weiss *et al.* (2022).

In this study, we utilized the abundance patterns from the aforementioned study, in which an *in vivo* metagenomic analysis of the infant mouse gut microbiome was conducted, to infer the interaction network among microbial strains. Specifically, we used the EDAME method (Fig. 10a) to infer the interaction network and then compared it to networks generated by SPIEC-EASI and SparCC (Supplementary Fig. S8b and c). The analysis performed here and in Weiss *et al.* (2022) indicate that most strain-strain interactions are competitive, with additional amensalistic interactions reported in Weiss *et al.* (2022). While the network in Weiss *et al.* (2022) is directed, networks were constructed undirected using all three methods.

For the purpose of comparison, the directed network from Weiss *et al.* (2022) was converted into an undirected form by representing amensalistic and parasitic interactions as competitive and commensalistic interactions as mutualistic. The three methods (EDAME, SPIEC-EASI, and SparCC) produced weighted, undirected networks. The Jaccard Index (JI) was then computed for positive, negative, and neutral links at various weight thresholds, using the original edges from Weiss *et al.* (2022) as a reference (Supplementary Fig. S9). The maximum JI achieved for each method is presented in Fig. 10b. The results demonstrate that all three methods exhibit comparable performance in edge detection.

It is noteworthy that the network in Weiss *et al.* (2022) (Supplementary Fig. S8a) was derived from *in vitro* pairwise co-culture and monoculture experiments. However, we were unable to utilize the abundance patterns provided for the *in vitro* study, as the number of unique attractors generated from these patterns was inadequate, resulting in erroneous results. Consequently, we used the *in vivo* abundance patterns from the same study. The authors of the study highlight the differences in species behaviour between pairwise co-cultures and complex community structures, specifically noting that all species except for ‘*Enterococcus faecalis* KB1’ were out-competed in at least one pairwise culture, while in the complex community structure, 10 out of 12 species were able to co-exist for up to 10 days. Despite the Jaccard Index for the network comparison being modest, the network generated (Fig. 10a) appears to preserve some of the key features highlighted in the paper, such as

- ‘*L. reuteri* I49 and *E. faecalis* KB1 dominated the small intestinal community in infant mice’—The high number of competitive links from I49 and KB1 to all other strains, along with a strong positive link between these two strains, suggests the dominant nature of both strains.
- ‘Notably, *B. coccoides* YL58 is involved in five of seven competitive interactions of the consortium’.—The competitive potential of YL58 is also clearly evident in the network.
- ‘Replicate communities showed reproducible community structure, even when different inocula were used. Interestingly, *F. plautii* YL31 dominated the community under these conditions’.—Similarly, the competitive potential of YL31 further supports this observation from the paper.

The strains *E. faecalis* KB1, *B. coccoides* YL58, and *F. plautii* YL31 exhibit dominant roles under various conditions. In order to accurately characterize their behaviour, it is essential to construct directed networks. The challenges associated with this approach, along with potential future directions, are discussed in Section 4.

## 4 Discussion and conclusion

With increasing interest in the microbiome, there is a high demand for tools to analyse microbial data and investigate relationships between different taxa. Here, we presented an algorithm to obtain an interaction network among microorganisms from their binarized abundance patterns, assumed to be attractors of the system. We believe the presence and absence of microbial species in a system contain the decisive information on network architecture and hence can explain the systematics of the system. The algorithm is built upon a simple principle that the network generated should have the same attractors as the original system. This was done by studying the sensitivity of attractors to small topological changes in the network. Iterating this principle, we evolve partially predicted networks to completely predicted ones, with the partially predicted networks obtained from the network inference method ESABO (Claussen *et al.* 2017). There are two main challenges faced by the EDAME algorithm:

- **Incomplete Attractors:** The prerequisite of *sufficient metagenomic sampling* is hard to fulfil. Despite the advances in sequencing techniques, it is still very expensive and not easy to obtain sufficient data. Due to limited metagenomic sequencing, for an underlying microbial interaction network, we do not obtain all possible attractors of the system. Since the algorithm works primarily by comparing attractors, the incomplete attractors become misleading for the algorithm. We anticipate that the following three-step strategy will allow us to address this challenge in our future work: (i) Calibrating the strategy from Mendler *et al.* (2024) for estimating the completeness of a dataset using simulated data and then applying it to a range of examples to see, whether datasets predicted to have a high degree of completeness lead to more similar inferred networks. (ii) Evaluating the relevance of basin size for the ESABO-based network inference using simulated data. We can assume that attractors with large basin sizes are more likely to be part of a given microbiome dataset. It is therefore a relevant question, whether a network inference based solely on attractors with large basins provides a reliable estimate of the true network. (iii) Leveraging the information from multiple phylogenetic levels to estimate the completeness of a microbiome dataset and to potentially augment the dataset by attractors likely to be part of the system based on the dynamics of the finer-grained substructure of each node.
- **Large networks**: The network correction method becomes computationally expensive as we move to large networks, since we perform exhaustive enumerating of attractors, i.e. finding attractors for all possible initial conditions. For a network of size N, the time complexity required to compute all possible attractors is $O(2^{N})$. The worst-case time complexity of the EDAME algorithm, excluding attractor enumeration, is $∼O(N^{5})$, primarily due to the calculation of all possible edge combinations $O(N^{4})$ within the network. However, our heuristic approach—where we first determine the sorted nodes array ($S_{N}$) and prioritize the most sensitive nodes to identify edge changes—significantly reduces the effective time complexity to less than $O(N^{5})$, though it cannot be precisely quantified. In certain cases, when the signal is not well-defined, the algorithm may need to evaluate up to the last node, which can pose a challenge for predictions in large networks Due to this, we restrict ourselves to small networks, hence to the phylum level in the applications to microbial data.

We believe focusing on the large basin attractors of the system could be a solution to both the challenges. As next steps, we will focus on understanding how informative attractors are as a function of their basin size.

Another way to avoid the computational load while exhaustively enumerating the attractors can be to use some existing network reduction methods that work by finding stable/oscillating network motifs (Zañudo and Albert 2013, Gan and Albert 2018) of the system. Making use of these techniques, which exist mainly for directed networks, is a promising direction of future research to bring the EDAME algorithm to a broader level of applicability (e.g. to more detailed phylogenetic levels; see below). In general, the restriction to undirected networks in our present investigation, which we imposed to keep the algorithmic description transparent, does not give us access to all unidirectional forms of interactions among microbial species, e.g. commensalism, parasitism, etc. This could be one of the reasons the attractor overlap does not reach a perfect score for real microbial systems. The EDAME algorithm can, in principle, work for both directed as well as undirected systems, but we need more tools to provide a fair initial prediction of directed networks. Methodologies like the one described in Nasrollahi *et al.* (2021) may pave the way towards such algorithmic extensions.

Due to the challenges above, we restrict ourselves to the phylum level of taxonomy in application of the algorithm to real data. Even though we see strong differences in health conditions on such a coarse level as well, it would be interesting to explore the differences on lower taxonomic levels. Once we have addressed the problem of incomplete attractor sets in our future work, we can then focus on the lower taxonomic levels. Distinguishing ‘true’ zeros in microbial abundance data from zeros arising from undersampling [‘zero-inflated microbiome data’; see, e.g. Pan (2021); Xia *et al.* (2018)] is a challenge particularly at finer phylogenetic levels. When extending EDAME to larger networks and incomplete attractors, this statistical property needs to be addressed. Consistency relationships among phylogenetic levels to estimate a level-appropriate binarization threshold may be a strategy to achieve this.

In this study, our main focus is microbial interaction networks, however, the algorithm is not limited to microbiome data and ESABO networks, and can be used with other network inference algorithms working under Boolean dynamics.

## Supplementary Material

btaf095_Supplementary_Data

## Data Availability

The data and code underlying this article are available on GitHub repository ‘edame’, at https://github.com/Jojo6297/edame.git.
