# Supplementary material for: Evaluating changes in attractor sets under small network perturbations to infer reliable microbial interaction networks from abundance patterns
Source: Bioinformatics. 2025 Mar 1;41(4):btaf095. doi: 10.1093/bioinformatics/btaf095 (PMC11961200; doi:10.1093/bioinformatics/btaf095)
Supplement: btaf095_Supplementary_Data [file btaf095_supplementary_data.pdf]

Evaluating changes in attractor sets under small network perturbations to infer reliable microbial interaction networks from abundance patterns

Jyoti Jyoti[1*], Marc-Thorsten Hütt[1]

[1] School of Science, Constructor University, Bremen gGmbH Campus Ring 1, Bremen, Germany

*Corresponding author:
Email: jjyoti@constructor.university

Marc-Thorsten Hütt
Email: mhuett@constructor.university

Supporting information

Appendices

Appendix S1

1. **Jaccard Index**: Measurement of agreement between two sets A and B, defined by:

$$JI(A,B) = \frac{|\, A \bigcap B \,|}{|\, A \bigcup B \,|}$$

$JI(A,B) = 0$ for disjoint sets, 1 for $A = B$.

2. **Hamming distance**: Hamming distance between vectors p and q of length m:

$$HD(p,q) = \sum_{i=1}^{m} \frac{1}{m}, \quad p[i] = q[i]$$

3. Quality Index: The Quality index is a quantity that represents the ratio of intra-disease-cohort network similarity to inter-disease-cohort network similarity.
QI excluding T2D and CRC from normalization:

$$QI(H, IBD) = \frac{Q_{HH} + Q_{II}}{2 * Q_{HI}}$$

QI including all:

$$QI(H, T2D, CRC, IBD) = \frac{2 * (Q_{HH} + Q_{TT} + Q_{CC} + Q_{II})}{(Q_{HT} + Q_{HC} + Q_{HI} + Q_{TC} + Q_{TI} + Q_{CI})}$$

where Q_{HI} = mean(Healthy-IBD compartment).

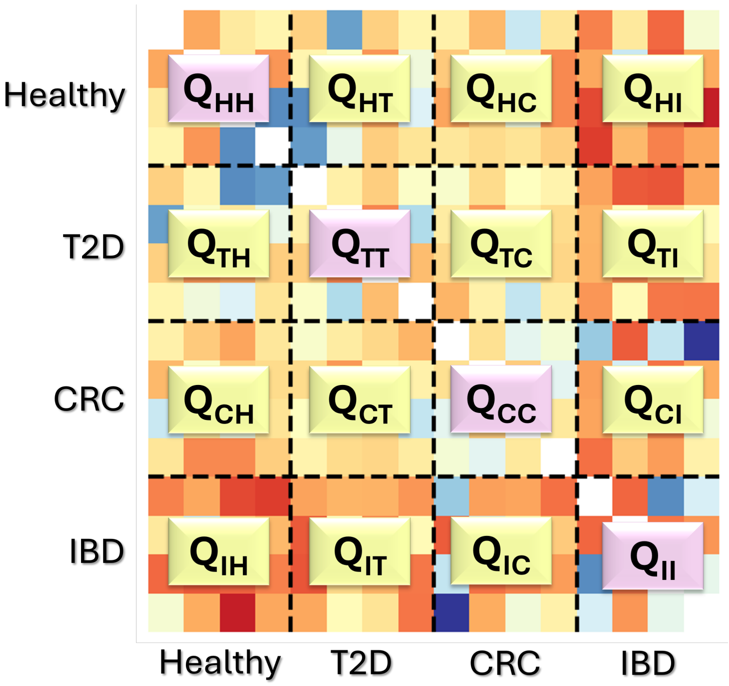

Fig A1: Heatmap representing the Jaccard similarity among the networks created for the four disease cohorts - healthy, T2D, CRC, IBD; $Q_{HI} = mean(JI$ among networks generated by healthy and IBD patients' samples .)

Appendix S2

Algorithm 1: EDAME (Edge detection via attractor mismatch evaluation)

```
   input  : G*, I*, A* and A
   output : Original network G
1  def PredictionProcess():
2      initialize fail ← 0
3      initialize iter ← 0
4      initialize JI_A ← 0
5      define P_C ← [(0,1), (−1,−1), (0,0), (−1,1), (1,1), (1,−1), (−1,0), (1,0), (0,−1)]
6      while JI_A < 1 do
7          Find the overlap between A and A*, JI_A ← JI(A, A*)  /* JI → Jaccard Index (Formula
           in Appendix S2) */
8          Find sort|E| for A and A*, as explained in Methods.
9          for i in range(2:N) do                          /* numbering starts from 0 */
10             for element in sort|E|[i : ] do
                   /* Select first i nodes from sort|E| and iteratively choose a node from
                   i+1 to N^th node, to create N_list, from which combinations of 2 nodes
                   form edges in E_list, further combinations of 2 edges form E_pair.  */
11                 N_list ← [sort|E|[0], ..., sort|E|[i − 1], element]
12                 E_list ← Combinations(N_list, 2)
13                 E_pair ← Combinations(E_list, 2)
14                 for (e1, e2) in E_pair do
15                     for comb in P_C do
16                         if I*[(e1)], I*[(e2)] = comb then
17                             for comb_edge in (P_C − comb) do
18                                 I*[(e1)], I*[(e2)] ← comb_edge
19                                 G_t ← network generated after modification
20                                 A_t ← attractor set of G_t
21                                 JI_t ← JI(A, A_t)
22                                 if JI_t < JI_A then
23                                     continue loop (while ignoring the steps below)
24                                 if JI_t > JI_A and JI_t ≠ 1 then
                                                               /* Accept the modification */
25                                     iter ← iter + 1
26                                     G* ← G_t
27                                     A* ← A_t
28                                     break out of all inner loops (start from line 7 with new G* and
                                       A*)
29                                 if JI_t = 1 then
30                                     iter ← iter + 1
31                                     G* ← G_t
32                                     A* ← A_t
33                                     break out of all loops
34             if i = N and JI_t ≠ 1 then
35                 fail ← fail+1
                   /* algorithm has failed, start the whole process again with randomized
                   E_pair                                                              */
36     G_end ← G*
```

SI Text

Evaluating the performance of sorted nodes array (S_N)

The data object S_N is a ranked list of nodes, obtained from the comparison of attractors of two networks, sorted in descending order with respect to the differences in attractors (see Figs 1,2). Here, we show that the highest-ranked nodes in S_N, i.e., the nodes which have highest attractor differences, indeed are predictive of the edge difference between the two networks.

Fig S2 summarizes the performance of S_N. In Fig S2a we recorded, how many initial nodes in S_N are required to find 2 nodes involved in 1 edge rewiring for 1000 independent experiments (500 positive, 500 negative edge changes) for random undirected networks with $N = 10$ and $M_+ = M_- = 20$. High density of points around the first 2 - 4 nodes on the y-axis shows that, in most cases, the first 2 - 4 entries in S_N suffice to predict the edge involved in rewiring. Note that the median for negative edge rewiring is lower than the median of positive edge rewiring. This suggests a higher sensitivity of attractors with respect to negative edges as compared to positive edges.

Fig S2b shows the positions of n_0, n_- and n_+ nodes, collectively, in S_N, with about 79% nodes getting detected in initial 5 nodes, and 62% in initial 3 nodes.

Temporary failure of the algorithm

The EDAME algorithm is based on evaluating the attractor mismatch between the original attractors and the partially inferred network's attractors, and using the information of varied nodes to repair edges in the intermediate network. But sometimes, the sequence of edge repairs takes a wrong path and reaches a point from where it is impossible to find an edge change increasing the attractor overlap further (failure of the algorithm). Attractor overlap does not always monotonically increase with network overlap between two systems. It is possible, after an iteration, to get an increased attractor Jaccard index, with a decreased network overlap. In this situation, the algorithm is terminated and started over with randomized edge pairs generated from S_N, to avoid the wrong turns on the path.

Inference of microbial phylum networks using co-abundance analysis

In this section, we generated networks for all cohorts (healthy, T2D, CRC, and IBD) at the species level using binarized abundance patterns to maintain consistency with the EDAME algorithm. Edges were defined based on significant ($p < 0.05$) Pearson correlations: positive for correlations > 0, negative for correlations < 0, and neutral (0) for non-significant correlations. To coarse-grain the species-level network to the phylum level, we summed all positive edges between species from two phyla and separately summed all negative edges. The resulting interaction network, representing the net positive or negative interactions between phyla, is displayed in Figure S11. Interestingly, we observed that most networks exhibit a high degree of edge overlap, irrespective of disease type (see Fig. S10). However, the clear segregation of disease types observed in the EDAME analysis at the phylum level is absent in this approach. Instead, network similarity does not appear to follow a specific pattern.

Supplementary figures

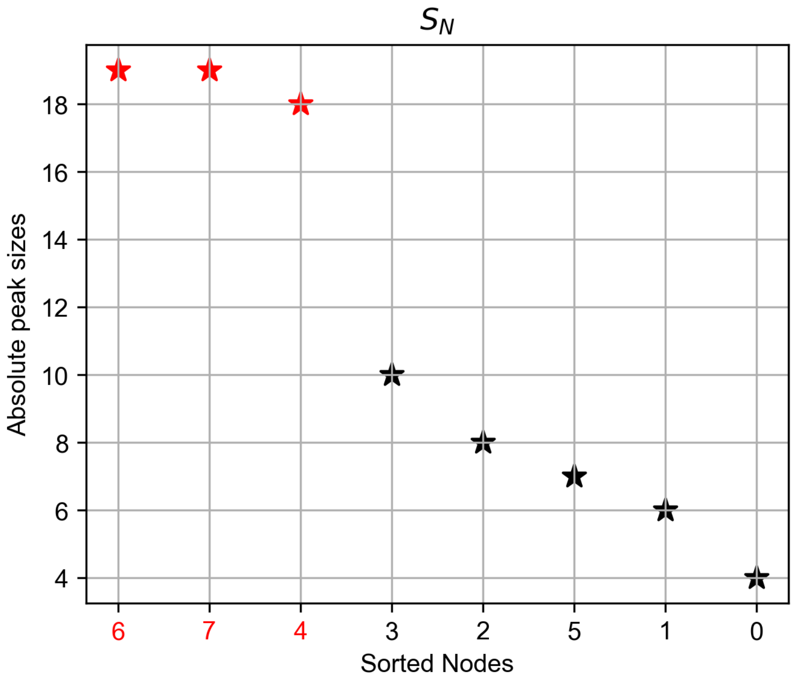

Fig. S1 S_N array representation.

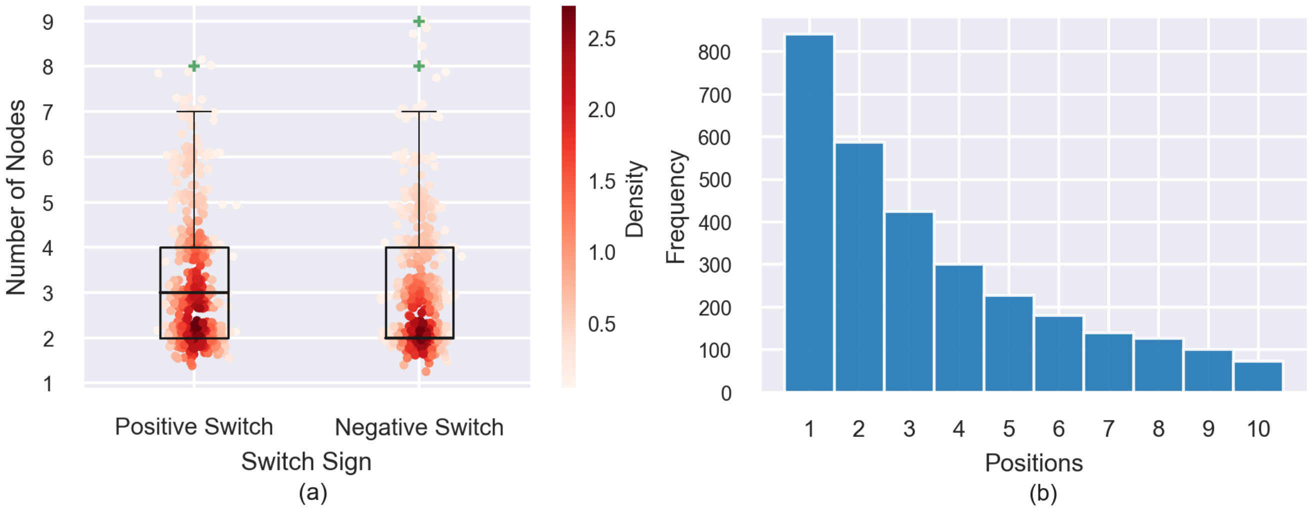

Fig. S2 (a) Scatter-box plot for the number of initial nodes in S_N required to predict an edge in rewiring. Points are randomly displaced by a small amount for better visibility of overlapping points. (b) Histogram depicting the positions of nodes in the rewired edge in S_N.

Performance of Algorithm: Modified edge prediction with ESABO

With failure limit = 50 and no iteration limit,

(a) Box plot showing an overlap of attractor sets A and A^* at the end of the algorithm. Overlap = 1 implies algorithm was successful, failed otherwise.

(b) Overlap of networks G and G_{end}.

(c) Box plot showing how many times the algorithm failed before succeeding (Failures > 50 excluded).

(d) The success rate for each edge difference, with the number of experiments with that particular edge difference displayed on the top of the bar.

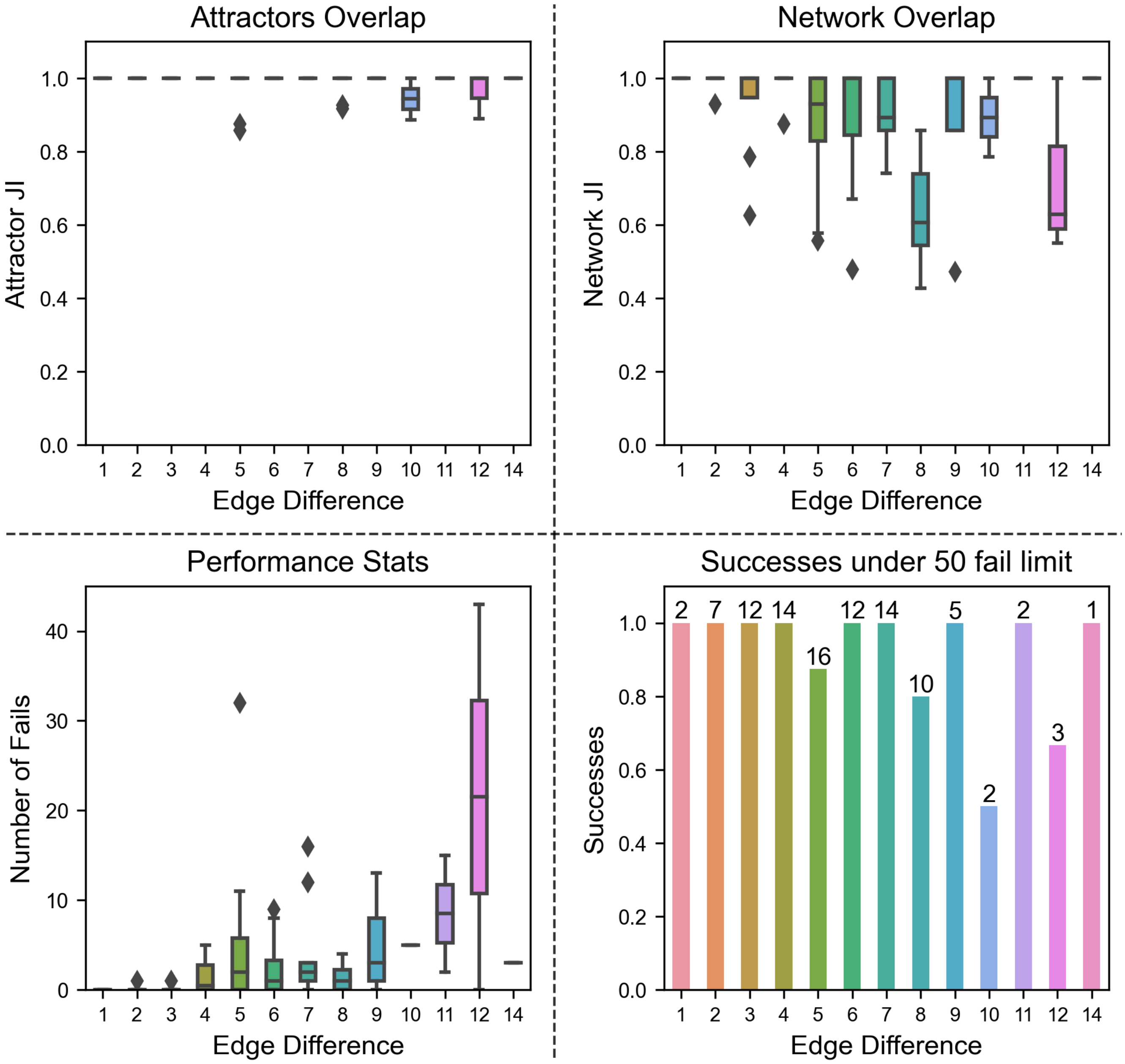

Fig. S3 $N = 8$, $M_+ = M_- = 6$, *x-axis: Remaining edge difference after ESABO prediction, that the algorithm tries to corrects for.*

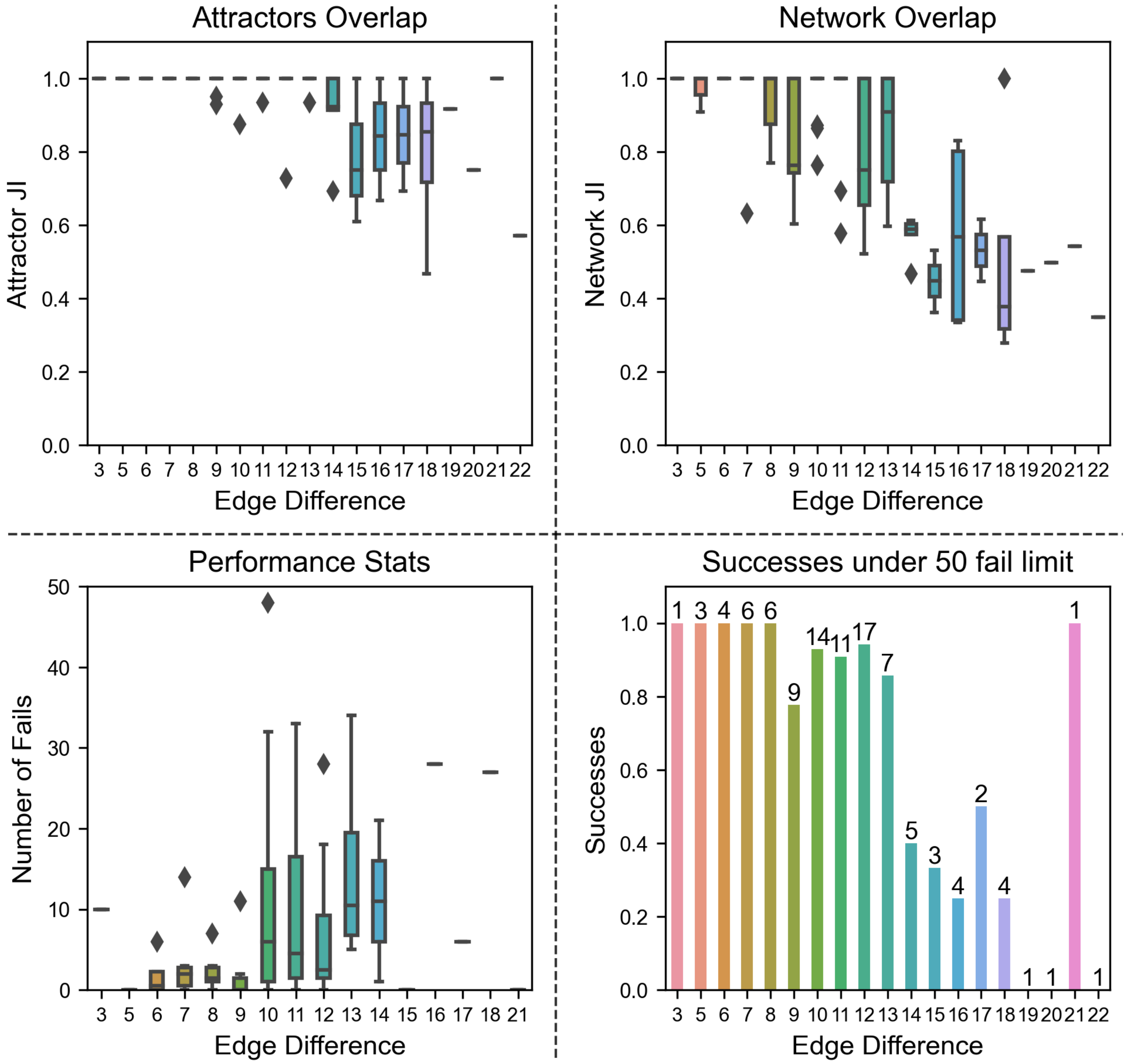

Fig. S4 $N = 10$, $M_+ = M_- = 10$, *x-axis: Remaining edge difference after ESABO prediction, that the algorithm tries to corrects for.*

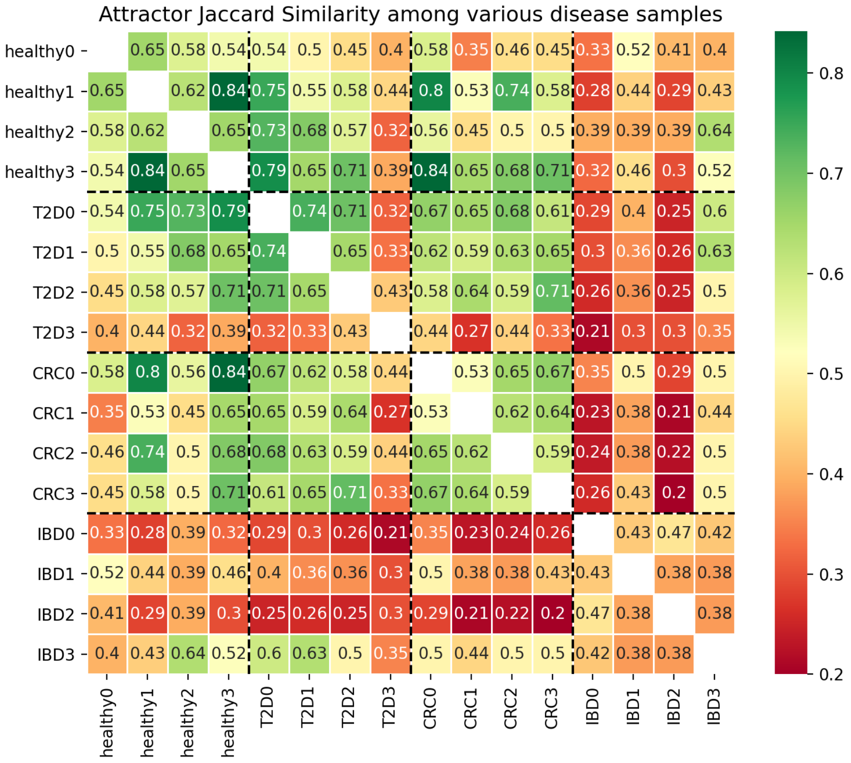

Fig. S5 Similarity among the abundance patterns of healthy, CRC, IBD samples.

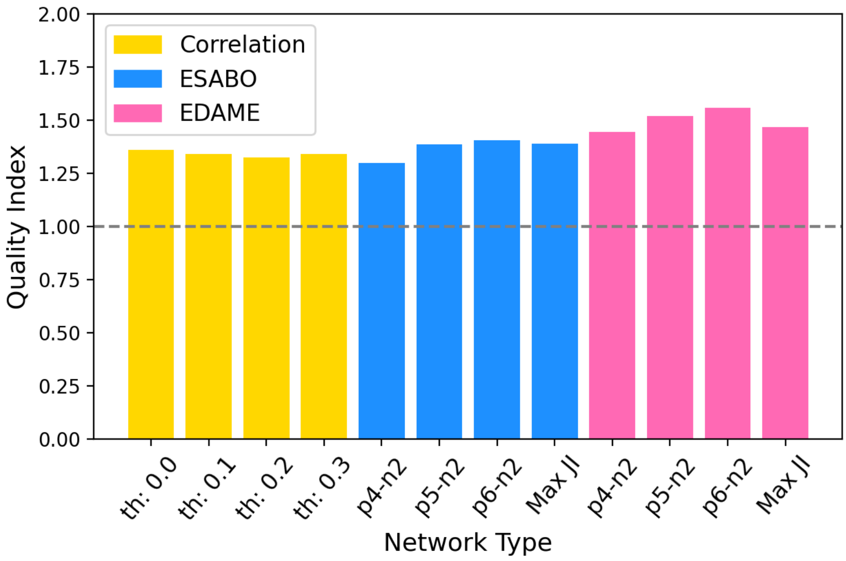

Fig. S6 Quality index (QI) plot with healthy, CRC, IBD data.

Comparison of similarity of networks within and across healthy, CRC, IBD cohorts generated by Correlation, ESABO, EDAME across various initial conditions.

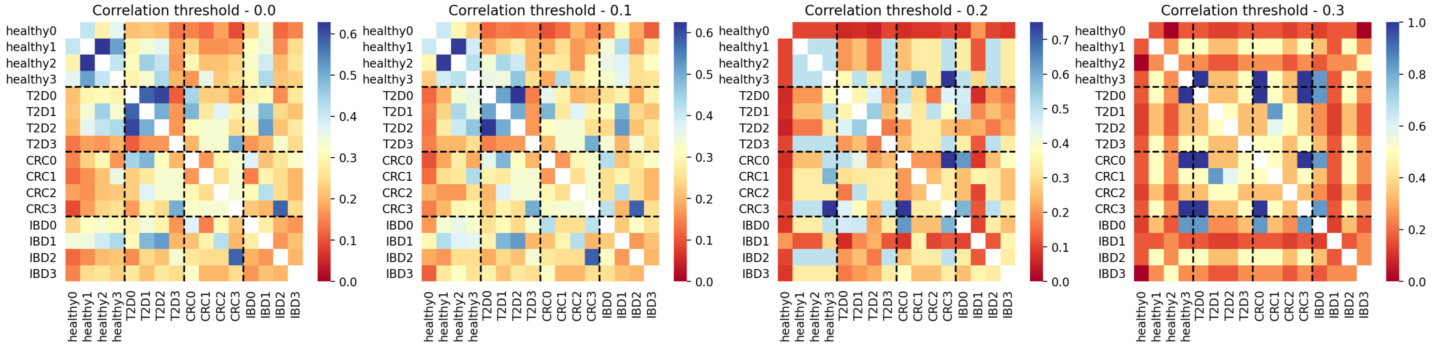

(a) Correlation networks with thresholds = {0, 0.1, 0.2, 0.3}

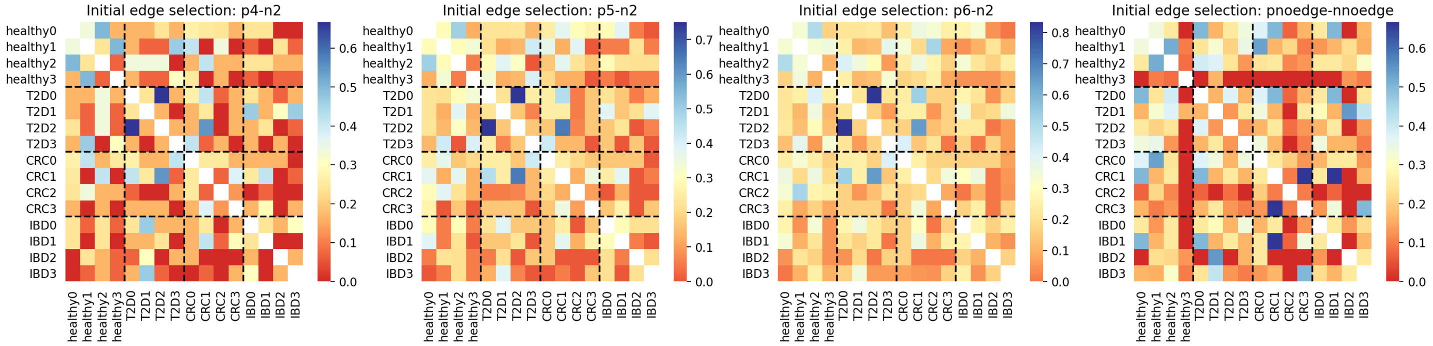

(b) ESABO networks with edge selection: positive edges = {4,5,6,random}, negative edges = {2,random}

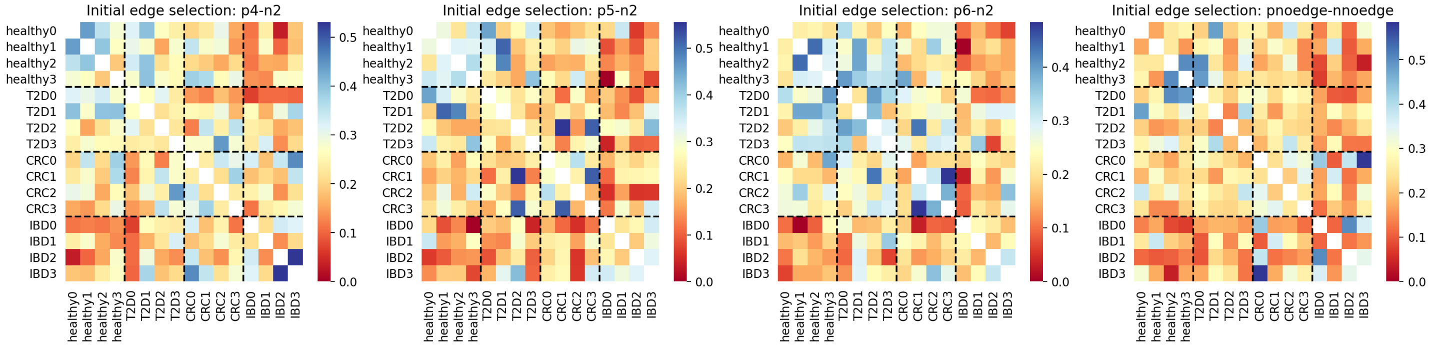

(c) EDAME networks with respective ESABO networks as initial points.

Fig. S7

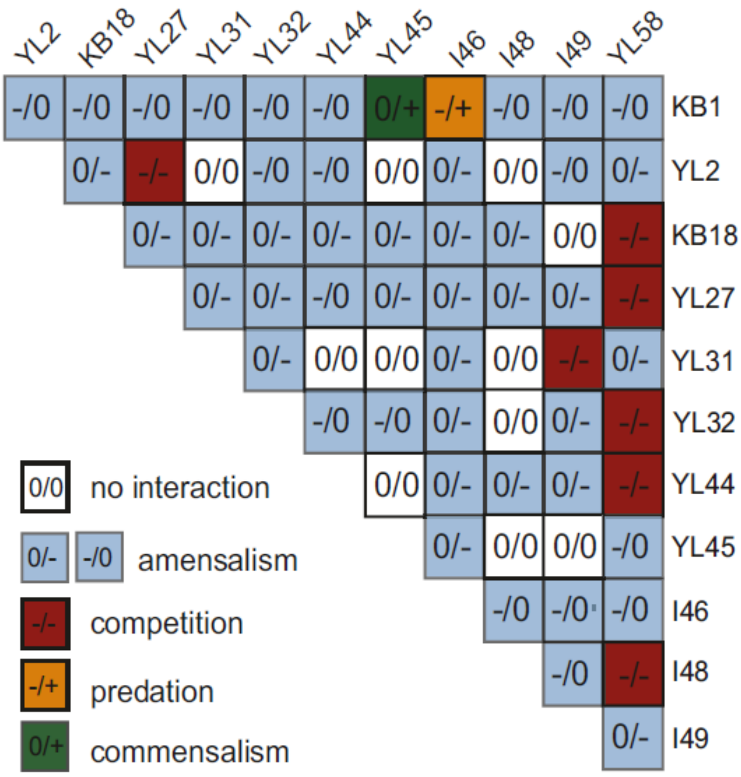

(a) The directed interaction network generated from comparison of pairwise co- and mono-cultures for OMM[12] bacterial strains from Weiss et al. 2022

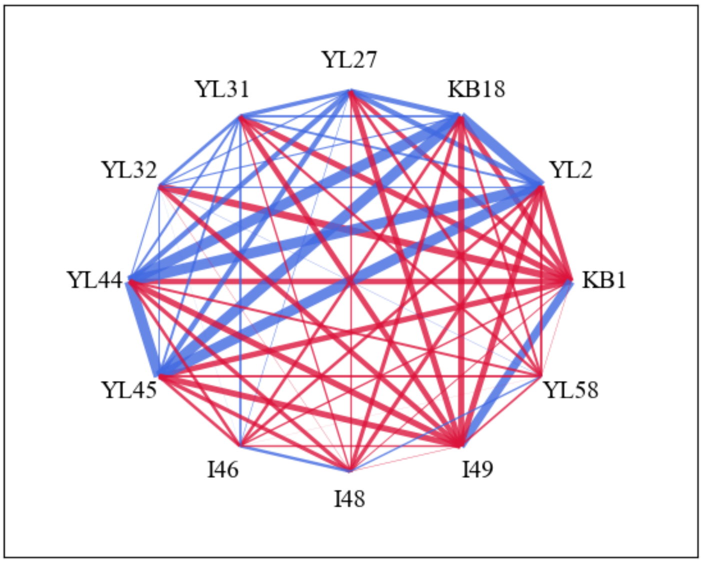

(b) Network inferred using SPIEC-EASY on the infant in vivo mice abundance patterns

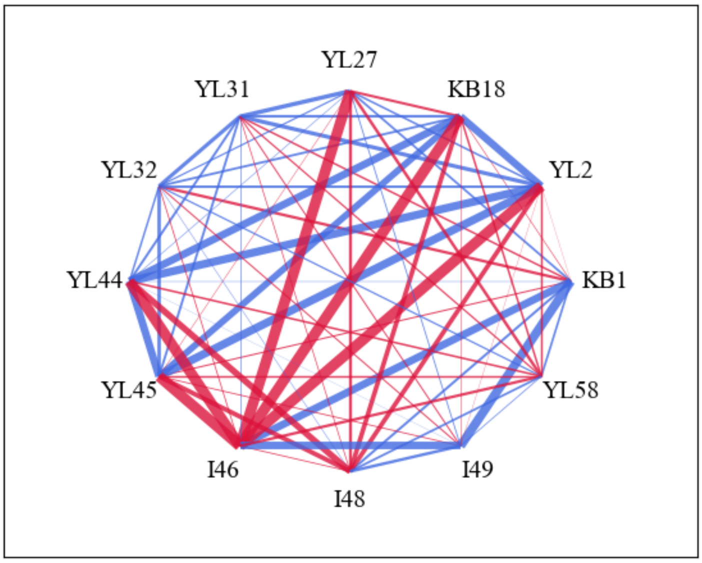

(c) Network inferred using SparrCC on the infant in vivo mice abundance patterns

Fig. S8

	positive-edge JI	negative-edge JI	no-edge JI
0.000000	0.000000	0.466667	0.000000
0.545055	0.000000	0.406780	0.090909
1.090110	0.000000	0.333333	0.206897
1.635165	0.000000	0.314815	0.222222
2.180220	0.000000	0.259259	0.225000
2.725275	0.000000	0.259259	0.222222
3.270330	0.000000	0.222222	0.212766
3.815385	0.000000	0.166667	0.211538
4.360440	0.000000	0.055556	0.183333
4.905495	0.000000	0.037037	0.174603

(a) **EDAME**

	positive-edge JI	negative-edge JI	no-edge JI
0.000000	0.000000	0.508197	0.000000
0.099802	0.000000	0.466667	0.058824
0.199604	0.000000	0.389831	0.071429
0.299406	0.000000	0.237288	0.125000
0.399208	0.000000	0.192982	0.155556
0.499009	0.000000	0.157895	0.137255
0.598811	0.000000	0.018519	0.150000
0.698613	0.000000	0.000000	0.147541
0.798415	0.000000	0.000000	0.145161
0.898217	0.000000	0.000000	0.145161

(b) **SPIEC-EASY**

	positive-edge JI	negative-edge JI	no-edge JI
0.000000	0.000000	0.433333	0.000000
0.100000	0.000000	0.350877	0.173913
0.200000	0.000000	0.214286	0.105263
0.300000	0.000000	0.125000	0.122449
0.400000	0.000000	0.125000	0.134615
0.500000	0.000000	0.111111	0.169811
0.600000	0.000000	0.092593	0.166667
0.700000	0.000000	0.092593	0.160714
0.800000	0.000000	0.092593	0.183333
0.900000	0.000000	0.092593	0.180328

(c) **SparCC**

Fig. S9 Comparing OMM[12] published network with the networks obtained from (a) EDAME, (b) SPIEC-EASY, (c) SparCC inference methods. The Jaccard Indices obtained on using different thresholds on the weights of edges. We consider the maximum from each edge type (positive, negative, neutral).

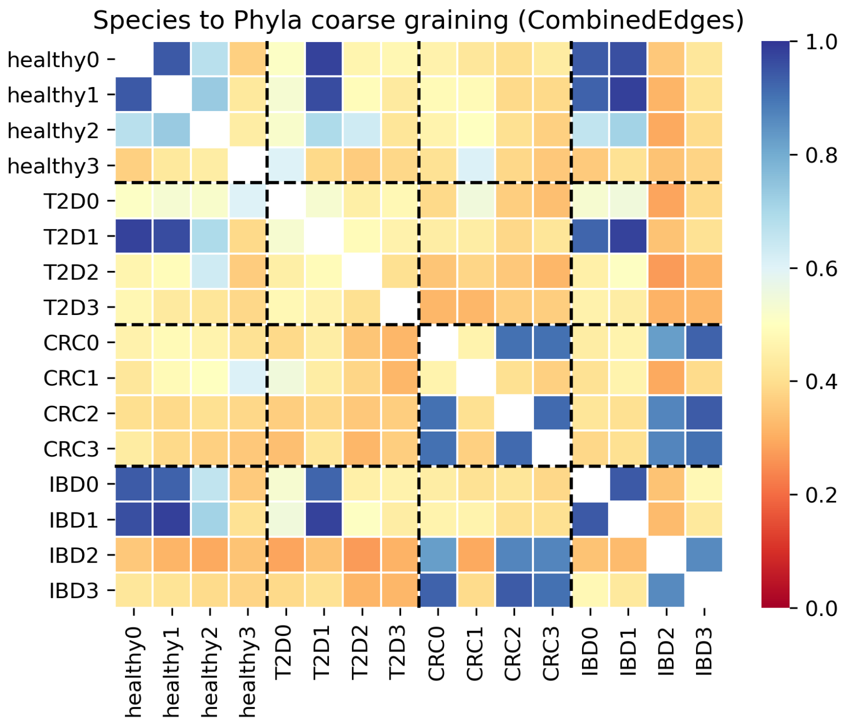

Fig. S10 Species to phyla level coarse grained correlation network heatmap

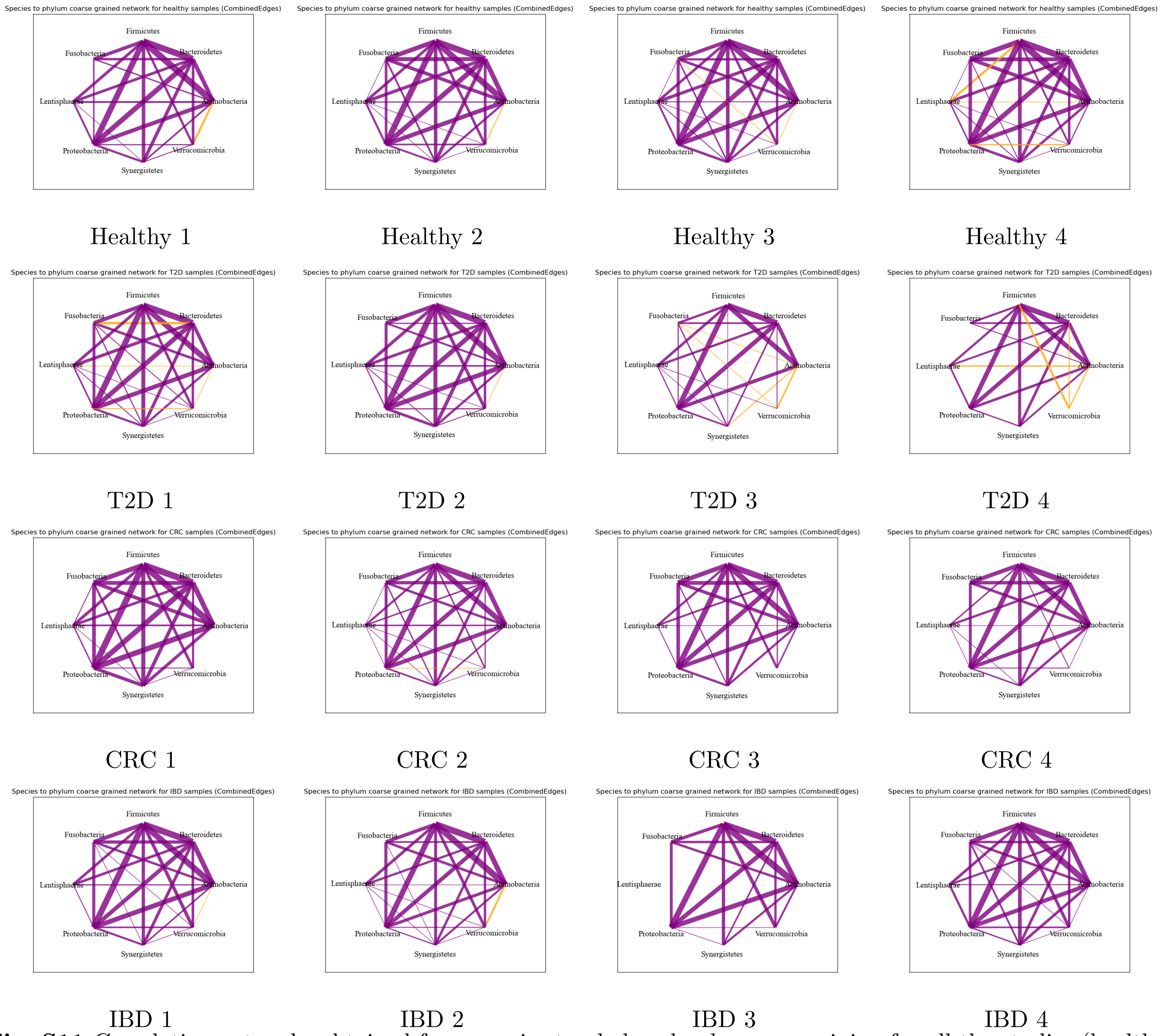

Fig. S11 Correlation networks obtained from species to phylum level coarse graining for all the studies (healthy, T2D, CRC, IBD); Purple = Net positive edge among the phyla, orange = Net negative edge among the phyla

References

Weiss, Anna S et al. (2022). “In vitro interaction network of a synthetic gut bacterial community”. In: *The ISME journal* 16.4, pp. 1095–1109.
